# Latency reversal agents affect differently the latent reservoir present in distinct CD4^+^ T subpopulations

**DOI:** 10.1371/journal.ppat.1007991

**Published:** 2019-08-19

**Authors:** Judith Grau-Expósito, Laura Luque-Ballesteros, Jordi Navarro, Adrian Curran, Joaquin Burgos, Esteban Ribera, Ariadna Torrella, Bibiana Planas, Rosa Badía, Mario Martin-Castillo, Jesús Fernández-Sojo, Meritxell Genescà, Vicenç Falcó, Maria J. Buzon

**Affiliations:** 1 Infectious Diseases Department, Hospital Universitari Vall d'Hebron, Institut de Recerca (VHIR), Universitat Autònoma de Barcelona, Barcelona, Spain; 2 Banc de Sang i Teixits, Hospital Universitari Vall d'Hebron, Universitat Autònoma de Barcelona, Spain; University of North Carolina at Chapel Hill, UNITED STATES

## Abstract

Latency reversal agents (LRAs) have proven to induce HIV-1 transcription in vivo but are ineffective at decreasing the size of the latent reservoir in antiretroviral treated patients. The capacity of the LRAs to perturb the viral reservoir present in distinct subpopulations of cells is currently unknown. Here, using a new RNA FISH/flow ex vivo viral reactivation assay, we performed a comprehensive assessment of the viral reactivation capacity of different families of LRAs, and their combinations, in different CD4^+^ T cell subsets. We observed that a median of 16.28% of the whole HIV-reservoir induced HIV-1 transcripts after viral reactivation, but only 10.10% of these HIV-1 RNA^+^ cells produced the viral protein p24. Moreover, none of the LRAs were powerful enough to reactivate HIV-1 transcription in all CD4^+^ T cell subpopulations. For instance, the combination of Romidepsin and Ingenol was identified as the best combination of drugs at increasing the proportion of HIV-1 RNA^+^ cells, in most, but not all, CD4^+^ T cell subsets. Importantly, memory stem cells were identified as highly resistant to HIV-1 reactivation, and only the combination of Panobinostat and Bryostatin-1 significantly increased the number of cells transcribing HIV within this subset. Overall, our results validate the use of the RNA FISH/flow technique to assess the potency of LRAs among different CD4^+^ T cell subsets, manifest the intrinsic differences between cells that encompass the latent HIV reservoir, and highlight the difficulty to significantly impact the latent infection with the currently available drugs. Thus, our results have important implications for the rational design of therapies aimed at reversing HIV latency from diverse cellular reservoirs.

## Author summary

HIV infection is an incurable disease. Despite antiretroviral therapy, a pool of cells with HIV in a latent state persists and precludes fully eradication of the viral infection. The cells that contain this latent viral reservoir are very diverse, and therefore different therapeutic strategies would be necessary to target and eliminate all infected cells. Latency Reversal Agents (LRAs) are compounds able to awake the latent virus from its dormant state with the purpose of making infected cells visible to the immune system. But the ability of the LRAs to target different cell types containing HIV is currently unknown. Here, using a novel methodology that interrogates individual cells, we found that current LRAs do not impact equally all infected cells. In fact, certain types of memory lymphocytes, recognized to harbor latent HIV for decades, are not fully impacted by most of the LRAs tested. Our study highlights the difficulty to cure HIV with the currently available LRAs. Different therapeutic approaches aimed at reversing HIV latency from diverse cellular reservoirs are needed to reduce HIV persistence.

## Introduction

Current antiretroviral therapy (ART) is extremely effective at suppressing HIV viremia below the limit of detection of standard clinical assays and substantially reduces the morbidity and mortality associated with the HIV-1 infection. However, ART is unable to fully eliminate and eradicate HIV from the human body [1,2]. This is mainly due to the presence of latently HIV-infected cells generated in the early stages of the infection that are not susceptible to current antiretroviral drugs [3]. The development of new clinical strategies targeting the persistent virus may lead to a long-term drug-free remission of HIV infection, which currently represents a high priority for HIV-1 research [4,5].

Over the last years, the “kick and kill” therapeutic strategy has been pursued as an approach for eliminating HIV; latently HIV-infected cells are pharmacologically forced to induce HIV transcription with the hope that viral reactivated cells will be cleared by virus-induced cytopathic effects or by the immune system [6–8]. In this regard, drug discovery efforts have identified several latency reversal agents (LRAs), compounds that can efficiently induce HIV expression. Vorinostat, Romidepsin and Panobinostat belong to the histone deacetylase inhibitor (HDACi) family. HDACi can suppress the histone deacetylases enzymes that enzymatically remove the acetyl group from histones, and as a consequence they induce gene expression. Importantly, HDACi successfully reactivated latent HIV in the first-in-human clinical trials [9–11]. Further, Disulfiram, a drug previously used to treat alcoholism, has been shown to increase HIV transcription in a subgroup of ART-suppressed patients after in vivo administration [12]. However, so far, none of the current LRAs tested in patients have proven to be effective at decreasing the size of the latent HIV reservoir. Other compounds, not yet tested in humans, have shown promising results ex vivo. In this sense, the PKC (protein kinase C) agonists Ingenol [13] and Bryostatin-1 [14] are involved in the PKC pathway, which plays an important role in cellular latency and reactivation of HIV via NF-κB (nuclear factor kappa-light-chain-enhancer of activated B cells) signaling and via P-TEFb (positive transcription elongation factor b). The bromo and extra terminal (BET) bromodomain inhibitor JQ1 [15] also reactivates HIV by its effect through the P-TEFb. Lastly, a novel family of LRAs has been identified; the benzotriazoles successfully increase viral transcription dependent on STAT5 phosphorylation [16].

An important issue for current and future clinical trials aimed at curing HIV infection through the administration of LRAs is to determine how effective these compounds are in fully reactivating the virus from all latently-infected CD4^+^ T cell subpopulations. The CD4^+^ T cell pool encompasses a heterogeneous population of cells defined by the differential expression of cell surface receptors associated with different stages of cell maturation, activation and differentiation [17,18]. These CD4^+^ T cell subpopulations include naive cells (T_NA_), stem cell memory (T_SCM_), central memory (T_CM_), transitional memory (T_TM_), effector memory (T_EM_) and terminally differentiated cells (T_TD_). As HIV transcription level and infection frequency differ by cell type [19–22], the characterization of the responses of the different CD4^+^ T cell subpopulations to pharmacological HIV reactivation will guide us on the design of more effective therapies aimed at reducing HIV persistence.

Currently, the most used assay for measuring the impact of LRAs on HIV reactivation is the quantification of intracellular HIV-1 RNA by conventional quantitative PCR assays [11,23–25]. Several other methodologies, as the quantitative viral outgrowth assay (qVOA), Tat/rev induced limiting dilution assay (TILDA) or the quantification of viral DNA, have also been used to characterize the action of different LRAs in patient samples [26–28]. Recently, a new assay that detects HIV reactivation using a dual staining protocol of the viral protein p24 has been described [29]. However, due to the low number of cells responding to the LRAs in patients, current assays require the use of large quantities of cells to accurately measure viral transcription. Furthermore, the detailed characterization of the different cell subsets responding to LRAs has been scarce so far, since it requires the previous isolation of the specific cell subsets under evaluation. In order to overcome these limitations, we have recently reported a novel RNA FISH/flow method, which is based on the quantification of viral RNA by flow cytometry allowing the quantification and phenotyping of cells expressing HIV-1 RNA molecules at the single cell level [30,31]. Importantly, HIV-1 RNA expressed in different subpopulations of CD4^+^ T cells can be successfully determined by this novel system.

Here, we have used and validated the RNA FISH/flow assay as a novel methodology suitable to evaluate compounds that can be pursued to reactivate the latent virus in patient-derived HIV infected cells. Using this methodology, we have characterized the specific responses of different CD4^+^ T cell subpopulations to the action of several LRAs families and their combinations. Overall, in CD4^+^ T cells we found that, on average, 16.28% of cells containing HIV-1 DNA were able to reactivate HIV with the most potent LRAs tested. From these cells, only a small fraction (~10%) produced the viral protein p24. Furthermore, we observed heterogeneous responses of specific cell differentiation phenotypes to these compounds, and we identified the combination of Romidepsin plus Ingenol as the most effective drug combination to efficiently increase HIV transcription and p24 production in most CD4^+^ T cell subsets. These findings highlight the difficulty to find LRAs able to reactivate HIV present in all cellular reservoirs; an essential requirement for the “kick and kill” therapeutic strategy to successfully impact persisting HIV in infected patients.

## Results

### HIV reactivation kinetics with single and combined latency reversal agents in the latently infected cell line J-Lat

In order to evaluate the potency and timing of the different LRAs at reactivating latent HIV, we initially tested them in the latently infected cell line J-Lat (clone 10.6), which contains integrated but transcriptionally competent HIV proviruses that express the green fluorescence protein (GFP) after viral reactivation. We evaluated the following families of LRAs: HDACi (Panobinostat and Romidepsin), PKC agonists (Ingenol and Bryostatin-1) and a bromodomain inhibitor (JQ1). Drugs were used at concentrations previously shown to be effective at reversing HIV latency [23,32,33]. To best measure HIV reactivation and in order to avoid the loss of GFP signal due to the cell death of highly viral-reactivated cells, we treated cells with the pan-caspase inhibitor Q-VD-OPh before the addition of the compounds. Previous results showed that J-Lat cells and CD4^+^ T cells from patients stimulated with Panobinostat expressed higher levels of GFP and HIV-1 RNA, respectively, when cells were simultaneously treated with the pan-caspase inhibitor (**S1A and S1B Fig**).

**Fig 1:**
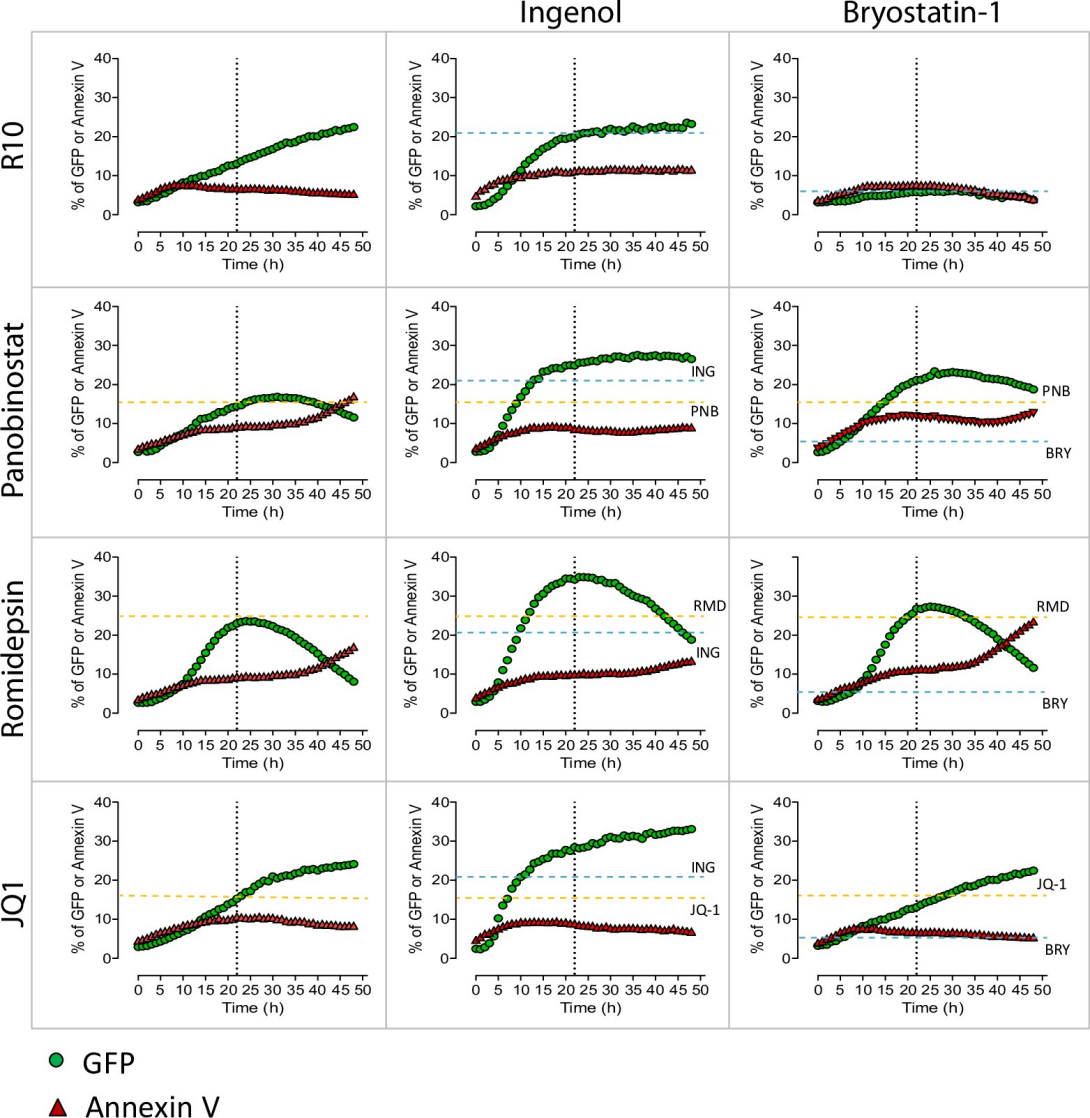
HIV reactivation kinetics in the latently infected cell line J-Lat. J-Lat cells were incubated for 50h with medium (R10), single LRAs or the combination of different families of these compounds; Panobinostat (PNB, 30 nM), Romidepsin (RMD, 40 nM), Ingenol (ING, 100 nM), Bryostatin-1 (BRY-1, 10 nM) and JQ1 (1 uM). Percentage of GFP^+^ (in green) and Annexin V^+^ (in red) cells was monitored each hour using the IncuCyte ZOOM live cell imaging system (Essen Bioscience). Dashed lines show the effect at 22h for the single drugs Panobinostat, Romidepsin and JQ1 (in yellow) and for Ingenol and Bryostatin-1 (in blue). Dotted lines represent 22h.

In J-Lat cells, after incubation with single LRAs and a detailed monitoring of the viral dynamics, we observed that viral reactivation for most LRAs tested reached its maximum or a plateau after 20-24h of drug exposure, except for JQ1 that gradually increased the expression of GFP during the entire incubation period (50h). Romidepsin induced the highest reactivation level (24% of GFP^+^ cells), followed by Ingenol and Panobinostat (20 and 15% of GFP^+^ cells, respectively) (**Fig 1**). In this model of latent infection, Bryostatin-1 did not induce a significant viral reactivation. In addition, we analyzed the effect that the combination of different families of LRAs had on HIV transcription. Romidepsin plus Ingenol was the most potent combination of LRAs, reaching values of 35% of GFP^+^ cells. Moreover, we also observed an additive effect at reactivating HIV when Ingenol was combined with Panobinostat and with JQ1 (27 and 28% of GFP^+^ cells, respectively). Importantly, in our experimental system, we observed that after 20-24h of drug treatment neither the single compounds nor their combinations induced more than 12% of cell apoptosis in J-Lat cells (**Fig 1**). Next, we tested the ability of the RNA FISH/flow assay to detect HIV-1 RNA and the viral protein p24 after the administration of LRAs. J-Lat cells were cultured with Romidepsin or with Romidepsin plus Ingenol for 22h (**S2A Fig**). We observed that, as previously shown [30,34], the RNA FISH/flow technology is able to distinguish two HIV-1 RNA positive populations: single HIV-1 RNA^+^ cells, and cells expressing both HIV-1 RNA and p24. Importantly, the population of cells expressing HIV-1 RNA and p24 was highly abundant (~90% of cells) when the culture was treated with the combination of the 2 LRAs, compared to single LRAs (~50% of cells) (**S2A Fig**). These values corresponded to percentages of GFP^+^ and HIV-1 RNA^+^ cells of 58.1% for Romidepsin alone and 90.2% for Romidepsin plus Ingenol (**S2B Fig**). Differences in the proportion of positive cells between these results and those provided in **Fig 1** are more likely due to the read out and the normalization method used to quantify viral reactivation with the different assays. Overall, we determined 22h as the more adequate timing to observe viral reactivation with all tested LRAs.

### Detection of HIV-1 RNA after viral reactivation with single and combined LRAs in primary CD4^+^ T cells

First, we measured drug toxicity induced by the addition of LRAs to primary CD4^+^ T cells. Early apoptosis, late apoptosis and cell death were identified as shown in **S3A Fig**. Overall, LRAs induced a maximum median of 11.34% of cell death (condition Romidepsin plus Ingenol) when drugs were added for 22h to previously-isolated CD4^+^ T cells obtained from uninfected donors (**S3C Fig**). However, under our experimental conditions, using the pan-caspase inhibitor Q-VD-OPh, no more than 3.04% of cell death was quantified (**S3B Fig**). Moreover, in CD4^+^ T cell subsets we observed that T_TD_ and T_EM_ cells, the more differentiated cell subsets, were more susceptible to cell death, especially when treated with the combination of Romidepsin and Ingenol (~10% dead cells, **S3D Fig**). On the contrary, T_CM_ and T_NA_ cells showed the lowest percentages of cell death when treated with different LRAs (maximum 2–3% of cell death) (**S3D Fig**). Drug toxicities in the absence of the caspase inhibitor are shown in **S3E Fig**. Of note, drug toxicity that remains despite Q-VD-OPh treatment might be caused by other cell death mechanisms, such as pyroptosis or cell necrosis [35]. Overall, Romidepsin plus Ingenol was the most toxic combination of drugs in all CD4^+^ T cell subpopulations. Nevertheless, even in this condition drug toxicity still remained relatively low after 22h of cell culture, and thus it is very unlikely that drug toxicity might preclude the interpretation of the viral reactivation assays.

**Fig 2:**
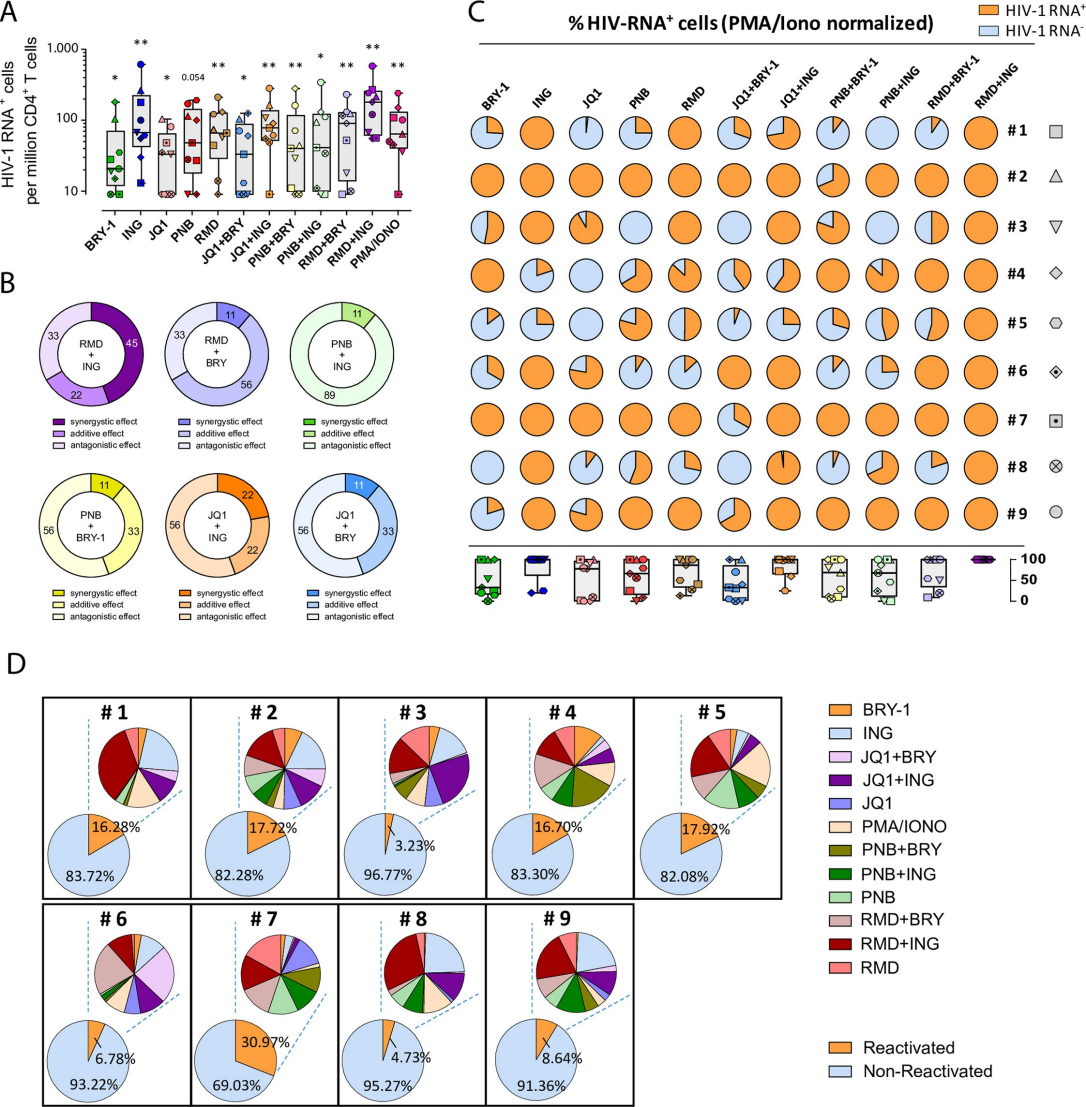
Detection of cells expressing HIV-1 RNA after viral reactivation with different LRAs. Freshly isolated CD4^+^ T cells from 9 ART-suppressed HIV-infected individuals were reactivated with different LRAs for 22h and subjected to the RNA FISH/flow protocol for detection of HIV transcripts. Drug concentrations were as follows: 40 nM Romidepsin (RMD), 30 nM Panobinostat (PNB), 1 μM JQ1, 100 nM Ingenol (ING), 10 nM Bryostatin-1 (BRY-1), 81 nM PMA plus 1 μM Ionomycin (IONO) or media alone. **A.** Proportion of cells expressing HIV-1 RNA in CD4^+^ T cells for each condition normalized to the medium control from individual patients are shown. Medians and min and max ranks are represented and statistical comparisons with the control medium were performed using the Wilcoxon test. *p<0.05, **p<0.01. **B.** Percentage of patients showing synergistic, antagonistic or additive effects (Bliss independence model) on HIV reactivation are shown as individual ring graphs for each combination of different LRA families studied. **C.** Proportion of HIV-transcribing cells relative to the positive control PMA/Ionomycin. Pies for individual patients normalized to the positive control and median values for all patients are represented in a box and whisker plot graph. HIV-1 RNA^+^ and HIV-1 RNA^-^ fractions are shown in orange and blue, respectively. **D.** Fraction of the HIV-reservoir susceptible to HIV reactivation after LRA treatment in CD4^+^ T cells from 9 ART-suppressed patients. Lower pies show the proportion of reactivated and non-reactivated cells. Upper pies show the fraction of reactivated cells after treatment with each compound depicted in the adjacent legend. We next calculated the percentage of the HIV-transcriptionally active viral reservoir after exogenous reactivation with the LRAs. We observed that between 3 and 31% (median value of 16.28%) of the total cells that encompass the viral reservoir (measured as the number of cells containing proviral DNA) were capable of transcribing HIV-1 RNA after viral reactivation (**Fig 2D**). The potency of each LRAs and their combinations in each individual patient is also shown in **Fig 2D**. Thus, while in general only a fraction of cells harboring HIV-1 DNA can be reactivated by current available LRAs, differences in terms of strength and consistency of this viral reactivation are observed in the whole population of CD4^+^ T cells from different ART-treated individuals.

Then, we tested the potential of LRAs to reactivate latently HIV-infected cells in fresh samples from 9 ART-treated individuals. At least 6x10^6^ isolated CD4^+^ T cells were cultured per condition during 22h, and a total of 13 conditions were set up for each patient. After viral reactivation, cells were subjected to the RNA FISH/flow assay in order to evaluate the frequency of cells that responded to the action of LRAs and were able to reactivate the latent provirus. The sensitivity of this assay at detecting HIV-1 RNA^+^ cells was previously established at 10 HIV-RNA^+^ cells per million cells [30]. The representative flow cytometry gating strategy used to identify HIV expression and production of the viral protein p24 in different subpopulations of CD4^+^ T cells is shown in **S4A Fig**. Overall, most tested LRAs and the combination of different families of LRAs significantly increased the frequency of cells expressing HIV-1 RNA in most patients compared to the non-reactivated control (p<0.05) (**Fig 2A**). For single LRAs, we observed that Ingenol, Romidepsin and Panobinostat increased the proportion of HIV-1 RNA expressing cells to higher levels (median values of 67 for Ingenol, 66 for Romidepsin and 48 for Panobinostat, expressed as cells per million, p = 0.0039, 0.0039 and 0.054, respectively, compared to the media control) than Bryostatin-1 or JQ1 (median values of 19 for Bryostatin-1 and 33 for JQ1, p = 0.015 and 0.039, respectively). However, only Ingenol was effective at reactivating HIV in all tested patients, compared to Romidepsin (8 out of 9 patients) and Panobinostat (7 out of 9 patients). For the combinations of LRAs, we observed that Romidepsin plus Ingenol promoted the highest induction of cells expressing HIV-1 RNA in all tested patients (p = 0.0039, fold change (FC) = 3.50, compared to the negative control) (**Fig 2A**). Of note, in this specific condition the proportion of HIV-1 RNA^+^ cells was even higher than the resulting proportion of cells cultured with the positive control of PMA and Ionomycin (median values of 180 for Romidepsin plus Ingenol and 64 for PMA and Ionomycin, p = 0.0078). We also observed that the combination of JQ1 with Ingenol induced a high proportion of cells expressing HIV-1 RNA (median value of 78, p = 0.0039) in 8 out 9 patients (**Fig 2A**). Furthermore, we calculated the synergistic, additive or antagonistic effects of the different combinations of LRAs using the Bliss independence Model [33,36]. We observed that Romidepsin and Ingenol presented the highest synergistic effect in 45% of the patients analyzed, while the other drug combinations promoted drug synergy in 22% of the patients at the most (**Fig 2B**). Moreover, it should be noted that the combination of Panobinostat and Ingenol induced an important antagonistic effect in 89% of the patients tested (**Fig 2B**). The synergistic effects between the different families of the LRAs in the individual patients are depicted in **S4B Fig**. Next, we normalized the values of RNA-expressing cells to the positive control, PMA and Ionomycin. Supporting the results showed in **Fig 2B,** we observed that Ingenol and Romidepsin were equally potent at reactivating cells expressing HIV than the positive control in 7 out of 9 and in 5 out of 9 patients, respectively. A more modest effect was observed for Panobinostat, Bryostatin-1 and JQ1 alone (**Fig 2C**). The best drug combinations again were Romidepsin plus Ingenol and JQ1 plus Ingenol, which increased the proportion of HIV-1 RNA expressing cells at levels comparable to the positive control in 9 out of 9, and 6 out of 9 patients, respectively. In addition, in patients #1, 3, 6 and 8, Panobinostat only reactivated at most half of the values obtained with the positive control. In the same patients, Ingenol was equally potent than the positive control; however, the combination of both drugs decreased the proportion of cells expressing HIV-1 RNA compared with the single drug Ingenol, compatible with an antagonist effect when both compounds are combined (**Fig 2C**). Besides, since we have recently described that CD4^+^ T cells expressing CD32^dim^ are enriched in HIV transcripts in vivo, and that viral infection upregulates this marker [30,37], we analyzed the expression of CD32^dim^ in viral-reactivated cells using different LRAs. We observed that CD32^dim^ was consistently upregulated after the pharmacological reactivation of HIV (**S4C Fig**).

### Detection of HIV protein p24 after viral reactivation with LRAs in primary CD4^+^ T cells from HIV-infected patients

**Fig 3:**
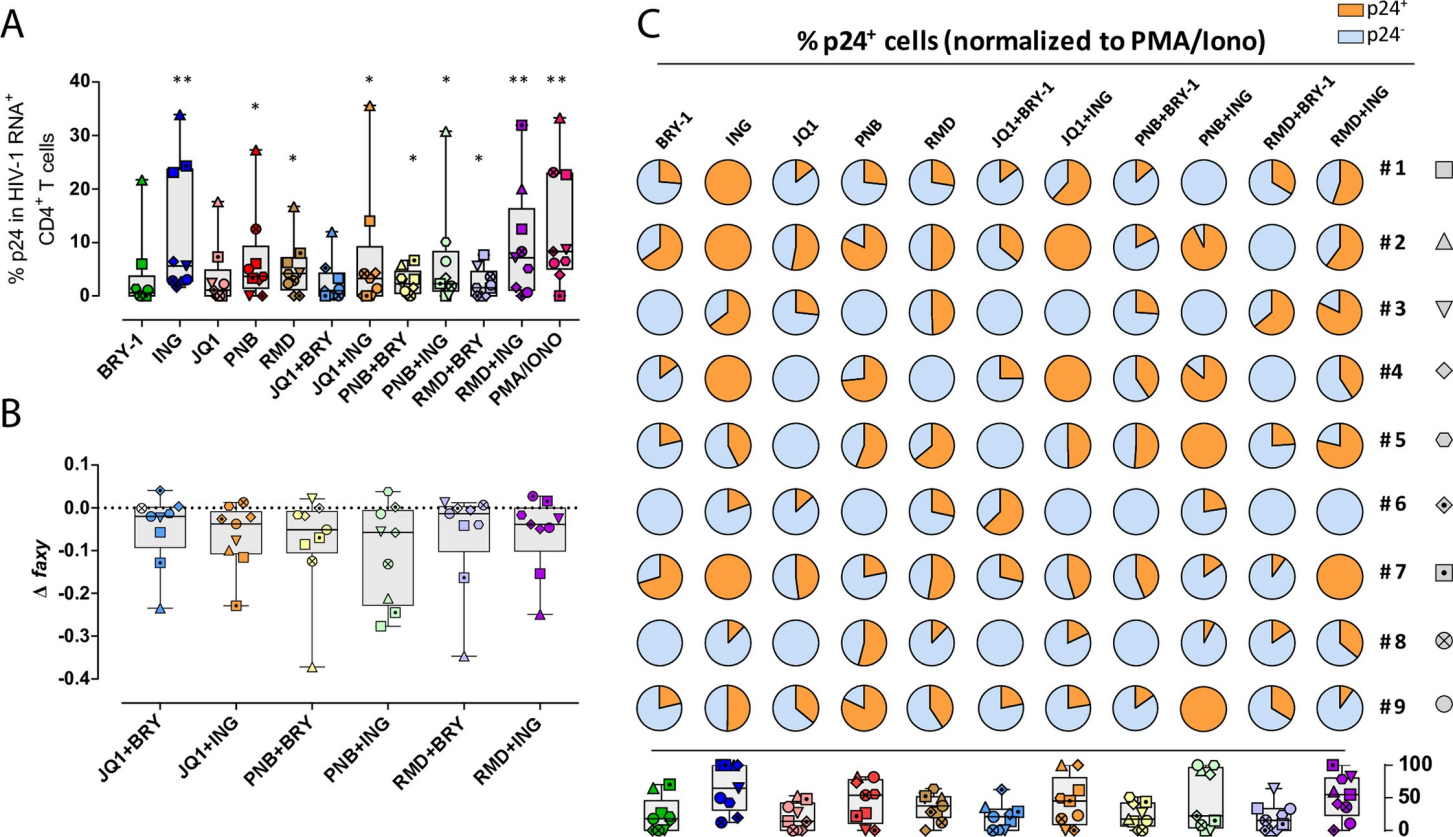
Proportion of CD4^+^ T cells expressing the viral protein p24 after viral reactivation with different LRAs. **A.** The proportion of HIV-transcribing CD4^+^ T cells that simultaneously produce the viral protein p24 is shown. Comparisons with the control medium were performed using the Wilcoxon test. *p<0.05, **p<0.01. **B**. Calculation of synergistic, antagonistic or additive effect after the combination of LRAs using the Bliss independence model. White symbols correspond to patients in which production of p24 was not detected. Medians and min to max ranks are represented in panels A and B. **C.** Normalization of the percentage of HIV-transcribing cells expressing p24 relative to PMA/Ionomycin. Pies for individual patients and median values for all patients are represented in a box and whisker plot graph. Fractions of HIV-1 RNA^+^ cells expressing p24 and lacking the expression of p24 are shown in orange and blue, respectively.

First, we determined the sensitivity of the assay at detecting HIV-1 RNA^+^ and p24^+^ cells. Primary ex vivo infected cells were spiked into uninfected cells at different ratios and the mixture was then subjected to the RNA FISH/flow protocol. We observed that dual expression of HIV-1 RNA and p24 determined by the experimental curve showed consistency with the predicted curve at all of the dilutions tested, establishing a limit of detection of 10–20 positive events per million cells (**S2C Fig**). Next, in order to determine whether LRAs and their different combinations were also capable of inducing the expression of the viral protein p24, we performed the RNA FISH/flow protocol with the simultaneous staining of intracellular p24. We analyzed the percentage of cells transcribing HIV-1 RNA that were also able to produce p24. As shown in **Fig 3A**, cells expressing p24 significantly increased after the addition of all LRAs but two, Bryostatin-1 and JQ1. Ingenol, Romidepsin and Panobinostat were the most potent LRAs at inducing the translation of viral transcripts (median values of 5.62, 4.17 and 3.64%, respectively). The combination of LRAs produced significant proportions of cells expressing p24, except when JQ1 was combined with Bryostatin-1 (median value of 0.99%). The positive control PMA and Ionomycin was the most potent drug (median value of 8.33%), followed by the combination of Ingenol and Romidepsin (median value of 7.14%, **Fig 3A**). However, in general, we did not detect more than 10% of HIV-1 RNA^+^ cells expressing p24. Of note, the combination of JQ1 and Ingenol produced high levels of cells transcribing HIV-1 RNA, however this combination only induced p24 in a modest proportion of cells (3.23%). Moreover, no synergies were detected for the production of the viral protein p24 in any of the combinations tested and, in concordance with the transcription data, we determined an antagonistic effect in the majority of patients when the combination of Panobinostat and Ingenol was used for viral reactivation (**Fig 3B**). Next, we normalized the proportion of p24^+^ cells to the maximum values obtained with the positive control (**Fig 3C**). In agreement with the results described in **Fig 2C**, we observed a negative effect when we combined Panobinostat and Ingenol (i.e patients #1 and 3) (**Fig 3C**). Furthermore, we observed a statistical significant correlation between the proportion of HIV-1 RNA^+^ cells and the percentage of the cells that are able to produce p24 after different LRA treatments (p<0.0001) (**S4D Fig**).

Taken together, individual LRAs have different capabilities of increasing the proportions of HIV-1 RNA and p24-expressing CD4^+^ T cells. In general, there was an agreement between the frequency of cells expressing HIV-1 RNA and p24, but some disconnection was observed for some LRAs; Romidepsin and Ingenol was the combination inducing the larger proportion of RNA-expressing cells, however PMA and Ionomycin outperformed them in their ability to induce p24-expressing cells. This is in agreement with previous reported results that determined that the positive control (anti-CD3/CD28 antibody-coated beads) was able to induce higher levels of multiply spliced Tat-Rev HIV-1 transcripts compared to unspliced HIV-1 RNA. On the contrary, Romidepsin induced higher levels of unspliced HIV-RNA compared to Tat-Rev transcripts [38]. Moreover, we detected an antagonist effect, in both HIV-1 RNA^+^ and p24^+^ cells, when Panobinostat and Ingenol were combined.

### Heterogeneous responses to LRAs of different CD4^+^ T cell subpopulations

**Fig 4:**
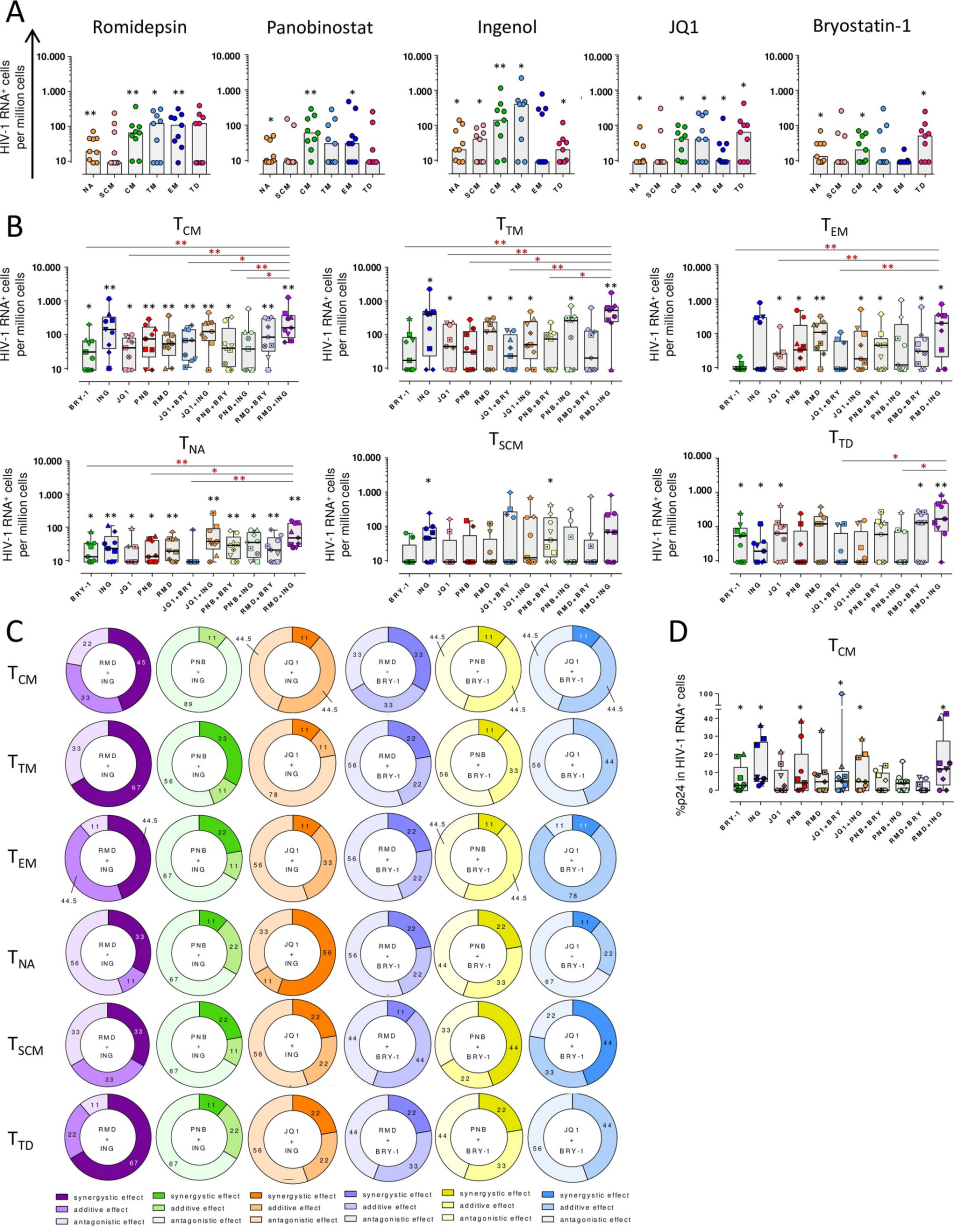
Proportion of cells expressing HIV-1 RNA and p24 after viral reactivation in different CD4^+^ T cell subpopulations. **A.** Proportion of cells transcribing HIV-1 RNA after viral reactivation with single LRAs in the following populations: T_NA_, T_SCM_, T_CM_, T_TM_, T_EM_ and T_TD_. Medians are shown. **B.** Proportion of HIV-1^+^ cells per million cells in each CD4^+^ T cell subpopulation with all tested drugs and their combinations. **C.** Proportion of patients showing synergistic, antagonistic or additive effects (Bliss independence model) after HIV reactivation with combinations of LRAs are shown for each CD4^+^ T cell subset. Percentage of patients responding to LRAs interactions are shown for each cell subset. **D.** Proportion of HIV-1 RNA^+^ T_CM_ cells expressing the viral protein p24. Black asterisks denote statistical significance compared with the negative control (media) using a Wilcoxon test. Red asterisks denote statistical significance compared with the combination of Romidepsin plus Ingenol using a Friedman test followed by Dunn’s post hoc tests. Medians and min to max ranks are represented in panels B and D. *p<0.05, **p<0.01.

Next, we focused our investigations on the capabilities of LRAs at reactivating HIV in different CD4^+^ T cell subpopulations. To do so, we isolated fresh CD4^+^ T cells from ART-treated patients and after LRA addition, transcription of HIV was measured by the RNA FISH/flow assay. Firstly, we assessed the impact of the different LRAs on the phenotypic markers used to identify the different CD4^+^ T cell subpopulations. We observe that the proportions of the different subsets were, in general, well maintained after treatment with the different drugs. Only very small differences were detected in some conditions (**S5A Fig)**. We consider that these changes are negligible and it should not significantly impact the proportion of virally-reactivated cells. Moreover, we observed that Romidepsin increased the proportion of memory cells transcribing HIV-1 RNA compared to the control, including central memory (T_CM_) (FC = 2.33, p = 0.0039), effector memory (T_EM_) (FC = 4.24, p = 0.007) and transitional memory (T_TM_) (FC = 4.06, p = 0.046) cells, and also naïve cells (T_NA_) (FC = 1.72, p = 0.0078) (**Fig 4A**). However, Panobinostat was less potent at inducing HIV-1 RNA^+^ cells in T_TM_; indeed, we observed the highest effect in T_CM_ (FC = 2.11, p = 0.0039) and a modest effect in T_EM_ and T_NA_ (FC = 2.32, p = 0.031 and FC = 1.27, p = 0.0156, respectively). Although significant, JQ1 induced a modest frequency of cells transcribing HIV-1 RNA in the majority of subsets analyzed, except for T_SCM_. Moreover, Ingenol preferentially reactivated T_CM_ (FC = 4.05, p = 0.0039) and T_TM_ (FC = 5.27, p = 0.0156) in most patients, which reached statistical significance. Although not significant, Ingenol also reactivated HIV in T_EM_ cells in 4 out of 9 patients. Finally, Bryostatin-1 reactivated very modestly some subsets, including T_NA_, T_TD_ and T_CM_ (FC = 1.49, p = 0.0156; FC = 1.94, p = 0.031; and FC = 1.22, p = 0.0156, respectively) (**Fig 4A**). A summary heatmap for the effect of single drugs is shown in **S6A Fig**. In addition, we analyzed the data stratified by CD4^+^ T cell subsets (**Fig 4B**). In general, T_CM_ and T_NA_ subpopulations were successfully reactivated by almost all tested drugs. T_CM_ and T_TM_ were more efficiently reactivated by Ingenol, while T_EM_ cells transcribed more HIV when cells were treated with Romidepsin. Moreover, HIV transcription was induced in T_NA_ cells more robustly after the addition of Ingenol and Romidepsin, and in T_SCM_ after Ingenol treatment. T_TD_ cells showed a distinct pattern of reactivation, since Ingenol, Bryostatin-1 and JQ1 were the only single LRAs that increased the proportion of cells expressing HIV in this specific subset (**Fig 4B**). Importantly, the combination of Romidepsin and Ingenol induced the largest proportion of cells transcribing HIV in most CD4^+^ T subsets, outperforming most LRAs and their combinations (FC = 3.44, p = 0.0039 for T_CM_; FC = 6.72, p = 0.0078 for T_TM_; FC = 6.96, p = 0.0156 for T_EM_; FC = 7.61, p = 0.0078 for T_TD_; FC = 2.54, p = 0.0039 for T_NA_; and FC = 2.61, p = 0.062 for T_SCM_) (**Fig 4B**). However, within the T_SCM_ subset only the combination of Panobinostat plus Bryostatin-1 was able to induce a significant increase of HIV-1 RNA^+^ cells (**Fig 4B**). A summary heatmap on the effect of drug combinations in the different CD4^+^ T cell subsets is shown in **S6B Fig**. Finally, we calculated the interactions between each drug combination in the individual patients (**S6C Fig**). In the majority of patients, the combination of Romidepsin and Ingenol was synergistic in memory cells (T_CM_ 45%, T_TM_ 67%, T_EM_ 44.5% and T_TD_ 67%) (**Figs 4C and S6C**). However, the combination of drugs that induced significant synergy in T_NA_ cells was JQ1 plus Ingenol (56%), and in T_SCM_ Panobinostat plus Bryostatin-1 (44%). As we already observed in the total CD4^+^ T cell population, the combination of Panobinostat and Ingenol produced an antagonistic effect in memory cells (T_CM_ 89%, T_EM_ 67%, T_TM_ 56%, T_SCM_ 67% and T_TD_ 67%). However, for the T_TM_ subset the combination of JQ1 and Ingenol was also antagonistic in most patients (78%) (**Figs 4C and S6C**). The reactivation induced by the positive control, PMA and Ionomycin, was not evaluated in CD4^+^T cells subsets due to the difficulty to gate accurately the different CD4^+^ T subpopulations.

Next, we investigated not only the capabilities of LRAs to induce HIV transcription, but also to generate the viral protein p24 expression in the different CD4^+^ T cell subsets. We observed that the combination of Romidepsin and Ingenol was able to induce a substantial increase of cells producing p24 in T_CM_ cells in most patients (**Fig 4D**), although it did not induce a synergistic effect (**S6D Fig**). Of note, for all remaining cell subsets, we were not able to detect significant number of cells expressing p24. The low percentage of cells expressing p24 and the low absolute number of HIV-1 RNA^+^ cells detected in the remaining subsets may explain this observation.

Overall, different CD4^+^ T cells subsets have different susceptibilities to LRAs and their combinations, but in general, we found that Romidepsin plus Ingenol was the most potent combination of LRAs, increasing significantly the proportion of HIV^+^ cells and producing a synergistic effect compared to the individual drugs. We also observed that T_CM_ and T_NA_ subpopulations presented broader susceptibility to the different families of LRAs, despite T_TD_ and, specially, T_SCM_ were more resistant to HIV reactivation. Furthermore, we determined a robust antagonistic effect when Panobinostat and Ingenol were used in combination in most subsets analyzed as we observed in the whole population of CD4^+^ T cells.

## Discussion

The elimination of the latently infected cell reservoir is believed to be the most important requirement to definitively eradicate HIV from the human body. Currently, therapeutic strategies named “kick and kill” are focused on the reactivation of these latent proviruses in ART-treated individuals using pharmacological compounds, the so-called LRAs. The ultimate goal of LRAs is to render infected cells susceptible to immune responses or to induce cell death by viral cytopathic effects. However, the impact of the drugs used to “kick” the virus on the subpopulations of cells that encompass the latent HIV reservoir is currently unknown. Importantly, clinical studies designed to perturb the latent HIV reservoir using LRAs have demonstrated an important increase in HIV transcription [10,11]. However, no effect was observed on the reduction of the size of the HIV reservoir following LRAs treatment. Since cell susceptibility to reactivating stimuli is the result of a complex interplay of individual viral and host factors, the inability of current drugs to reactivate latent HIV present in specific subsets of long-lived cells might help to explain the recent failures of clinical trials.

In this study, we investigated for the first time the impact of several families of LRAs, such as the HDACi (Romidepsin and Panobinostat), the PKC agonists (Ingenol and Bryostatin-1), and the bromodomain inhibitor JQ1, on their ability to induce viral transcripts in freshly-isolated CD4^+^ T cells from aviremic ART-treated HIV-infected individuals. Using a novel flow cytometry technique, the RNA FISH/flow method, we studied the reactivation of HIV from cellular reservoirs. Although we do not detect all fully replication-competent viruses, positive cells detected using this method are able to produce elongated HIV-1 RNA upon LRA treatment, and in some cases, to produce the viral protein p24. Importantly, the frequency of blood cells induced to transcribe HIV-1 RNA has been previously correlated with the frequency of cells induced to express infectious virus [39]. Therefore, detection of HIV-1 RNAs have biological importance.

Here, we observed that the fraction of the total HIV-reservoir that can be reactivated by the tested LRAs varied between patients, ranging from 3 to 31%. These results concur with Banga et al. [25], which indicated that only a fraction (≈2.6%) of HIV-1 proviruses were reactivated to produce virions, supporting also results from Cillo et al. [24]. This low percentage of viral reactivation might be explained, in part, by the fact that most of HIV-1 DNA present in ART-treated patients contains fatal mutations; only 2–10% of proviruses are considered intact, and therefore, more likely to produce full length HIV-1 RNA transcripts [40,41]. Moreover, between 60–80% of the proviruses might present deletions in the gag-pol region [40], precluding the detection of viral transcription by the RNA FISH/flow method. It should be note that we are using fresh CD4^+^ T cells from a single blood draw, and therefore it is uncertain if similar results will be obtained from blood collected at different time points. We speculate that the results using LRAs with cells collected at different time points will mainly depend on the size and stability of the transcriptionally-inducible HIV-1 reservoir. Of note, we have not measure the half-live of the transcriptionally-inducible HIV reservoir in ART-treated patients, but as demonstrated for other reservoir measurements, the stability will be more likely dependent on the individual patient; i.e. the half-live of the replication competent HIV reservoir is approximately 44 months (if measured as IUPM or IDPA) [40], but other factors, such as clonal expansion, ongoing viral replication, and redistribution of infected cells from lymphoid tissue, will significantly affect its longitudinal stability [42]. Additionally, the intrinsic stability of the different cell subsets that contain the inducible HIV-1, might also account for the variation in the level of viral reactivation.

For all tested drugs we observed a significant increase in the proportion of HIV-1 RNA^+^ cells, but some LRAs were especially potent at increasing their frequency. As single drugs, and in concordance with our in vitro data generated in the J-Lat cell line, Romidepsin and Ingenol induced the highest frequency of cells expressing HIV-1 RNA in nearly all samples. These findings are in agreement with previous reports; Pandeló et al. showed that Ingenol disrupted HIV latency at higher levels compared to PMA or Vorinostat, both in latency cell models and in infected primary resting cells [13], while Wei et al. demonstrated that Romidepsin induced an important increase in HIV-1 transcription compared to Vorinostat in both total memory and resting cells from HIV infected patients [43]. Moreover, we observed that JQ1 and Bryostatin-1 reactivated HIV-1 very poorly. In contrast to our results, it has been previously showed that in resting CD4^+^ T cells from HIV-patients only Bryostatin-1 induced an increment in the production of RNA compared to the HDAC inhibitors Romidepsin, Panobinostat and Vorinostat, and the bromodomain inhibitor JQ1 [23]. In the study, the authors used qPCR to measure viral reactivation, thus a potent induction of HIV in a limited number of cells might help to explain the discrepancy between both studies. Another study from Jiang et al. [44], described a synergistic effect when JQ1 plus Ingenol were combined, but in our work we observed an antagonistic effect in the 56% of the patients. The fact that different Ingenol molecules can be used for viral reactivation studies, the different methods used to detect viral reactivation, and the discrepancies observed between cell lines and primary CD4^+^ T cells in viral reactivation protocols [32], might explain this contradictory result.

Furthermore, we characterized the responses of each CD4^+^ T subpopulation to different LRAs. These investigations have rarely been performed before, mainly due to the difficulty to obtain enough cells from each CD4^+^ T cell subset to comprehensively quantify viral reactivation. In order to overcome this limitation, we used the novel RNA FISH/flow methodology that allows the simultaneous detection of HIV-1 RNA transcripts and the viral protein p24 at the single cell level without the need to previously isolate the fraction of cells being evaluated [30]. In general, each LRA was impacting differently the CD4^+^ T cell subpopulations; even drugs belonging to the same family had a differential effect on the same cell subsets. For instance, Panobinostat successfully reactivated HIV in T_CM_ cells, whereas Romidepsin was capable of impacting all memory cells (T_CM_, T_TM_ and T_EM_). Importantly, it has been shown that both drugs have different capacity to inhibit cell-associated HDAC activity [43]. Thus, it is tempting to speculate that differential expression of HDAC isoforms within different CD4^+^ T cell subsets could be associated to their intrinsic capability to reactivate latent HIV. In concordance with our results, in a recent study, cells treated with Panobinostatt that reactivated HIV appeared to be long-lived whereas Romidepsin appeared to reactivate HIV in shorter life span cells [45]. This study calculated the life span of cells that reactivated HIV in vivo using mathematical models. Consistently, Banga et al. showed that Panobinostat was slightly more robust than Romidepsin at reactivating HIV in isolated resting memory CD4^+^ T cells, a fraction enriched in long-lived central memory cells [25]. We also observed that T_CM_ and T_NA_ cells have the broadest susceptibility to the different families of LRAs, and Ingenol was extremely efficient at reactivating T_NA_, T_SCM_, T_CM_ and T_TM_ but did not show a significant effect on T_EM_ cells. In this regard, a recent study determined that the majority of cells expressing HIV-1 RNA in the presence of Ingenol had a T_CM_/T_TM_ and T_EM_ phenotype [31]. Additionally, while the T_NA_ subpopulation has not traditionally been considered as a cellular HIV reservoir, this subset has been recently described as a large inducible cell reservoir of both latent and replication competent virus at levels similar observed in T_CM_ [46], which is in concordance with our results. One of the main limitations of the present study is our inability to detect p24 in most of the cell subsets. In general, there was an agreement between the frequency of cells expressing HIV-1 RNA and cells producing p24. However, we were only able to detect p24 in the whole CD4^+^ T cell population and in T_CM_ cells. This is not the result of a poor sensitivity of the RNA FISH/flow method (10–20 positive cells per million), instead it might be explained by the low absolute number of HIV-RNA^+^ cells observed within subsets that were represented in small frequencies as i.e. T_SCM_, T_TM_, T_EM_ and T_TD_ (all below 30%).

We observed that long-lived cells such as T_CM_ or T_SCM_, previously defined to be important in the long-term maintenance of HIV reservoirs in patients [19,20], have different susceptibilities to the LRAs tested. For instance, T_SCM_ were poorly reactivated with most drugs. Only the combination of Panobinostat and Bryostatin-1, and to a lesser extent Ingenol, were able to significantly increase the proportion of HIV-1 RNA^+^ cells in this subset. This finding highlights the difficulty to identify LRAs with a mechanism of action broad enough to reactivate latent HIV present in all HIV-infected cells. Moreover, the lack of effect of the LRAs on T_SCM_ cells is a concern, since viral recrudescence from these long-lived cells might significantly preclude the in vivo long-term efficacy of LRAs tested in clinical trials. Thus, our results have important implications for rational design of therapies aimed at reversing HIV latency; the knowledge of the individual mechanisms that lead to viral reactivation in the population of cells that encompass the latent HIV reservoir will help with the development of LRAs with which to impact HIV persistence.

Importantly, we found that the combination of Romidepsin and Ingenol induced the highest frequency of HIV-1 RNA^+^ cells, even more than the positive control with PMA and Ionomycin, and this finding was consistent in all tested samples. To our knowledge, the combination of Romidepsin plus Ingenol has never been explored before in this setting. The independent mechanism of action of both drugs is most likely the responsible for the high number of HIV-1 RNA^+^ cells detected. This argument is supported by the observation that the combination of both drugs does not induce higher number of HIV-1 RNA molecules per cell (mean fluorescence intensity) (**S5B Fig**), instead it induces a broader spectrum of cells that are able to express HIV-1 RNA upon viral reactivation. Moreover, the synergistic effect was particularly evident in the T_CM_ and T_TM_ memory subsets, indicating that the differentiation or maturation status of the cells may be a critical determinant for a successful viral reactivation with the different LRAs. In this sense, it has been recently reported that CD4^+^ T cell subsets have distinct transcriptional profiles that are related to the level of HIV-1 infection and might modulate the response to external stimulus [47]. We also determined a robust antagonistic effect (89% in whole CD4^+^ T cells) when Panobinostat and Ingenol were combined. This is in agreement with the study presented by Larragoite et al. [48], in which they showed that the co-treatment with both drugs inhibited the reactivation of HIV in an ex vivo model of resting CD4^+^ T cells isolated from aviremic patients, despite a synergistic relationship was demonstrated in an in vitro latency cell model (J-Lat 10.6). ​The authors speculate that the inhibition induced by Panobinostat of the chaperone heat shock protein 90 (Hsp90), which is directly involved in the reversion of HIV-1 latency by Ingenol [49], might reduce the activation of the NF-kB pathway caused by the PKC agonist. This could explain the antagonistic effect observed when these two drugs are combined. In addition, it has also been observed that Panobinostat induces latency reversal by an Hsp90 independent way [48]. ​Further, these results manifest again the existing discrepancies between latently infected T cell lines and primary cell models of HIV-1 latency [32].

In conclusion, this study highlights the inability of current LRAs to fully reactivate HIV hidden in diverse cellular reservoirs. The identification of compounds with a broader reactivation capacity or the use of complementary drugs with different mechanisms of action will be needed to reactivate latent virus present in different cell types, where more likely diverse cellular pathways are implicated in silencing HIV.

## Materials and methods

### Ethics statement

PBMCs (peripheral blood mononuclear cells) from adults (>18 years old) HIV-1-infected patients were obtained from the HIV unit of the Hospital Universitari Vall d’Hebron in Barcelona, Spain. Written informed consent was provided by all patients who participated in this study, and the protocols used were approved by the Comitè d’Ètica d’Investigació Clínica (Institutional Review Board numbers 39–2016 and 196–2015) of the Hospital Universitari Vall d’Hebron, Barcelona, Spain. All samples were obtained only from adults and were totally anonymous and untraceable.

### Study samples

Samples from HIV-1-infected patients under ART with CD4^+^ T cell counts higher than 100 cells/mm^3^ and viral load <50 cop/ml for a mean (min-max) of 3 (1–6.5) years were recruited in the HIV unit of the Hospital Universitari Vall d’Hebron in Barcelona (Spain) and were included in this study. Information on plasma viral loads, CD4^+^ T cell counts, and time on ART for treated patients is summarized in **S1 Table**.

### Cells

Fresh PBMCs were obtained from a whole blood donation (400ml) from HIV-infected patients by Ficoll-Paque density gradient centrifugation and cells were immediately used without previous cryopreservation. Isolated CD4^+^ T cells (MagniSort Human CD4^+^ T Cell Enrichment; eBioscience) were cultured in RPMI medium (Gibco) supplemented with 10% fetal bovine serum (FBS; Gibco), 100 μg/ml streptomycin (Capricorn Scientific) and 100 U/ml penicillin (Capricorn Scientific), (R10). The human latently infected cell line J-Lat (clone 10.6) was obtained through the NIH AIDS Reagent Program from Eric Verdin [50]; cells were grown in R10 and maintained at 37°C in a 5% CO_2_ incubator.

### Viral reactivation with latency reversal agents

Isolated CD4^+^ T cells were stimulated during 22h with latency reversal agents (LRAs) at the following concentrations: 40 nM Romidepsin (Selleckchem), 30 nM Panobinostat (Selleckchem), 1 μM JQ1 (Sigma-Aldrich), 100 nM Ingenol-3-angelate (Sigma-Aldrich), 10 nM Bryostatin-1 (Tocris Bioscience), the positive control (PMA 81 nM plus Ionomycin 1 μM, both from Abcam), or the negative control (media alone, R10). Drugs were used at concentrations previously shown to be effective at reversing latency in studies performed in CD4^+^ T cells from HIV-infected individuals as well as studies performed in latency models in vitro [23,32,33]. All compounds were reconstituted in DMSO at the maximum concentration of 0.006%. Moreover, in order to prevent cell death induced by the reactivation of HIV and to evaluate the reactivation effect without confounding variables, cells were pre-treated with a pan-caspase inhibitor named Q-VD-OPh (quinolyl-valyl-O-methylaspartyl-[-2,6-difluorophenoxy]-methyl ketone, Selleckchem) [51,52]. Q-VD-OPh is a potent inhibitor for caspases 1, 3, 8 and 9, which are involved in the intrinsic and extrinsic apoptotic pathways, inhibiting consequently the specific cell death induced by HIV [53–56]. In all experiments, cells were treated with 10 μM of Q-VD-OPh for at least 2h prior to the addition of the latency reversal agents.

### Detection of viral reactivation and cell death by the IncuCyte Live-Cell analysis technology

HIV reactivation and toxicity effects induced by the different LRAs were longitudinally and exhaustively determined in the latently infected cell line J-Lat 10.6. Viral reactivation and cell death was monitored using the IncuCyte ZOOM live cell imaging system (Essen Bioscience). The latently infected cell line J-Lat contains integrated but transcriptionally latent HIV proviruses, in which the reporter gene GFP replaces the *nef* coding sequence [50]. GFP was used to measure viral reactivation and the apoptotic marker Annexin V (Essen Bioscience) was used to determine cell death induced by the drugs or by cytopathic effect. Briefly, cells were pre-treated with the pan-caspase inhibitor Q-VD-OPh for at least 2h and then seeded at 25.000 cells per well in a 96 well plate. Single LRA or combination of different families of LRAs were added to the corresponding well and Annexin V reagent (1:200) was immediately added on cells, with a final well volume of 100μl. Images were captured every hour for 48h from 2 independent wells per condition. Green (HIV expression) and red (Annexin V) object counts per well (1/mm^2^) were quantified at each time point and values were normalized to the confluence of each well.

### Drug toxicity assays in CD4^+^ T cells

Cell toxicity was assessed for single drugs and the combination of different LRA families in previously isolated CD4^+^ T cells from three independent uninfected donors. CD4^+^ T cells were pre-incubated with the pan-caspase inhibitor Q-VD-OPh for 2h. Afterwards, CD4^+^ T cells (200,000 cells per well) were incubated for 22h with the compounds. Then, cells were stained with the apoptotic marker Annexin V (PE, Biolegend) and a viability dye (LIVE/DEAD fixable Violet Dead Cell Stain kit, Invitrogen) in order to identify the following stages of cell death: live cells (Annexin V^-^ Viability^-^), early apoptotic cells (Annexin V^+^ Viability^-^), late apoptotic+necrotic cells (Annexin V^+^ Viability^+^) and total cell death (Annexin V^-^ Viability^+^). In addition, different surface markers, including CD3 (Pe-Cy7, BD Biosciences), CD4 (AF700, BD Biosciences), CD45RO (BV605, Biolegend) and CD27 (FITC, Biolegend), were used to identify drug toxicity induced in the different CD4^+^ T cell subpopulations. The CD4^+^ T cell subsets were identified as follows: Naïve (T_NA_) and Stem Cell Memory (T_SCM_) (CD3^+^CD4^+^CD27^+^ CD45RO^-^), Central (T_CM_) and Transitional Memory (T_TM_) (CD3^+^CD4^+^CD27^+^ CD45RO^+^), Effector Memory (T_EM_) (CD3^+^CD4^+^CD27^-^ CD45RO^+^) and Terminally Differentiated cells (T_TD_) (CD3^+^CD4^+^CD27^-^ CD45RO^-^).

### RNA FISH/flow assay of single cells expressing HIV-1 RNA transcripts and p24 protein after viral reactivation

PBMCs from nine ART-treated HIV-infected patients were obtained from a whole blood donation (400ml) and CD4^+^ T cells were isolated by negative selection using magnetic beads (MagniSort Human CD4^+^ T Cell Enrichment; eBioscience). A total of 13 conditions were assayed per patient and at least 6x10^6^ of freshly-isolated CD4^+^ T cells were subjected to viral reactivation per condition, which included the individual LRAs, the combination of 2 different families, and the positive and negative controls. Prior to viral reactivation, cells were pre-incubated with the pan-caspase inhibitor Q-VD-OPh for 2h. In order to block new rounds of viral infection during viral reactivation, cells were treated with LRAs in the presence of Raltegravir (1uM) during 22h. Afterwards, cells were subjected to the RNA FISH/flow protocol for the detection of HIV transcripts and the viral protein p24 following the manufacturer’s instructions (Human PrimeFlow RNA Assay; eBioscience) with some modifications, as previously described [30]. Briefly, PBMCs were stained with antibodies against cell surface markers and viability dye. Cells will be then fixed, permeabilized, and intracellularly stained for detection of the viral p24 protein. After an additional fixation step, cells will be ready for 3h of probes hybridization at 40±1°C with a high-sensitivity target-specific set of 50 probes spanning the whole Gag-Pol HIV mRNA sequence (bases 1165 to 4402 of the HXB2 consensus genome). The cells will be then subjected to different amplification steps (sequential 2h incubations at 40°C). Finally, multiple label probes will be hybridized with the specific amplifiers (1 h at 40°C) and samples will be run on an LSR Fortessa four-laser flow cytometer (Becton Dickinson).

In these experiments, to identify the different CD4^+^ T cell subpopulations expressing HIV-1 RNA and the viral protein p24, the following antibodies were used for cell surface staining: CD3 (AF700, Biolegend), CCR7 (Pe-CF594, BD Biosciences), CD27 (FITC, BD Biosciences), CD45RO (BV605, Biolegend) and CD95 (Pe-Cy5, BD Biosciences). The CD4^+^ T cell subset phenotypes were identified as follows: T_NA_ (CD3^+^ CCR7^+^ CD45RO^-^ CD27^+^ CD95^-^); T_SCM_ (CD3^+^ CCR7^+^ CD45RO^-^ CD27^+^ CD95^+^); T_CM_ (CD3^+^ CCR7^+^ CD45RO^+^); T_EM_ (CD3^+^ CCR7^-^ CD45RO^+^ CD27^-^); T_TM_ (CD3^+^ CCR7^-^ CD45RO^+^ CD27^+^) and T_TD_ (CD3^+^ CCR7^-^ CD45RO^-^). The surface marker CD32 (Pe-Cy7, Biolegend) was also included in the analysis. The expression of HIV-1 RNA transcripts was analyzed with target-specific AF647-labelled probes, and the expression of the Gag p24 viral protein was detected with a PE-anti-p24 antibody (clone KC57 RD1; Beckman Coulter). Cell viability was determined using a violet viability dye for flow cytometry (LIVE/DEAD fixable Violet Dead Cell Stain kit, Invitrogen). All values of HIV-1 RNA were normalized to the negative control (R10) corresponding to the non-reactivated cells from each patient.

### Sensitivity of the assay at detecting productive HIV-infected cells

To test the sensitivity of the assay at detecting cells expressing both HIV-1 RNA and p24, primary infected CD4^+^ T cells from HIV-infected patients were expanded. We used the same protocol described for the qVOA assay [30], and the positive wells were mixed up and diluted into uninfected cells at six different ratios. Samples were then subjected to the RNA FISH-flow assay. The predictive curve was determined by the basal expression of HIV-1 RNA and p24 and the subsequent theoretical values of the serial dilutions. The infection rate (experimental curve of percent HIV RNA^+^p24^+^ cells) was calculated by using the values obtained with the RNA FISH-flow assay. Linear regression was computed to determine the linearity of the relationship between the predicted and experimental values of the assay.

### Proviral HIV-DNA quantification by qPCR

CD4^+^ T cells were isolated by negative selection as mentioned above. For proviral quantification, 1 million CD4^+^ T cells were immediately lysed in a Proteinase K-containing lysis buffer (at 55°C over-night and at 95°C for 5 minutes). Cell lysates were subjected to HIV-DNA quantification by qPCR using primers and probes specific for the 1-LTR HIV region (LTR forward 5’-TTAAGCCTCAATAAAGCTTGCC-3’, LTR reverse 5’-GTTCGGGCGCCACTGCTAG-3’ and probe 5' /56-FAM/CCAGAGTCA/ZEN/CACAACAGACGGGCA/31ABkFQ/ 3'), as previously described [57]. CCR5 gene was used for cell input normalization. Samples were analyzed in an Applied Biosystems 7000 Real-Time PCR System.

### Statistical analysis

Statistical analyses were performed using the Prism software (GraphPad) version 6.01. Data are shown as the median and the min-max rank. Comparisons among the frequency of HIV-1 RNA expressing cells between unstimulated control (R10) and viral-reactivated conditions were performed using the Wilcoxon signed rank test. For correlations, Spearman’s correlation coefficient was calculated. To test the linearity of the assay, a linear regression was performed. A Friedman ANOVA test was used to compare the frequency of HIV-1 RNA^+^ cells induced by Romidepsin plus Ingenol with the levels induced by the other drugs in the different CD4^+^ T cell subsets, with corrected p-values for multiple comparisons (Dunn’s test). A p value of <0.05 was considered statistically significant. Synergies and antagonisms effects between drugs were calculated using the Bliss independence model (values <-0.09 were considered as highly antagonistic, values > 0.09 were considered as highly synergistic. Intermediate values between 0.09 and -0.09 were considered to have an additive effect). Data are presented as the difference between the observed and the predicted responses of each combination (∆f_axy_ = f_axy,O_—f_axy,P_), where f_axy,O_ is the observed fraction affected and f_axy,P_ is the predicted fraction affected. The f_axy,P_ is calculated as f_axy,P_ = f_ax_+f_ay_−(f_ax_·f_ay_) where f_ax_ is the fraction affected by drug X and f_ay_ is the fraction affected by drug Y [33].

## Supporting information

S1 Fig Q-VD-OPh inhibits the apoptosis of viral-reactivated cells. The effect of the pan-caspase inhibitor Q-VD-OPh on the detection of viral-reactivated cells was evaluated in isolated CD4^+^ T cells from ART-suppressed HIV-infected patients and in the latently infected cell line J-Lat (clone 10.6). **A**. GFP expression in J-Lat cells was monitored by the IncuCyte ZOOM live cell imaging system (Essen Bioscience) every hour for 50 hours after the addition of Panobinostat (30 nM) with or without Q-VD-OPh. **B.** CD4^+^ T cells were reactivated with Panobinostat (PNB, 30 nM) in the presence of Q-VD-OPh (10μM). Control cultures were treated with media alone (R10) or treated with media and Panobinostat (PNB). Copies of HIV-1 RNA per million CD4^+^ T cells were quantified in samples from 6 HIV^+^ patients by qPCR. Open circles show values under the limit of detection. Fold-change (FC) of viral reactivation is compared between conditions, but only in those where HIV-1 RNA was detected.
(TIF)

S2 Fig Detection of HIV-1 RNA and p24 after viral reactivation by the RNA FISH/flow assay in J-Lat cells. Cells were incubated for 22h with medium alone (R10), Romidepsin (RMD, 40 nM) or Romidepsin (40 nM) plus Ingenol (ING, 100 nM). Cells were then subjected to the RNA FISH/flow protocol and the proportion of HIV-1 RNA^+^ and p24^+^ (**A**) and HIV-1 RNA^+^ and GFP^+^ (**B**) cells was determined by flow cytometry. A flow cytometry plot for each condition is shown. **C.** Infection of primary CD4^+^ T cells from HIV-infected patients were expanded in vitro, and infected cells were diluted with uninfected cells to perform the quantification of predicted (blue symbols) versus experimental (orange symbols) values of HIV-1 RNA^+^ p24^+^ expression measured by the RNA FISH/flow assay. Assay linearity was assessed by linear regression.
(TIF)

S3 Fig Drug toxicities in CD4^+^ T cells and in CD4^+^ T cell subpopulations. Isolated CD4^+^ T cells from 3 uninfected donors were incubated with the different drugs for 22 hours (40 nM Romidepsin, 30 nM Panobinostat, 1 μM JQ1, 100 nM Ingenol, 10 nM Bryostatin-1, 81 nM PMA plus 1 μM Ionomycin or media alone) and cell death was evaluated by flow cytometry in the whole CD4^+^ T cell population and in the different CD4^+^ T cell subsets. Cell subsets were identified as Naïve and Stem Cell Memory (T_NA_/T_SCM_) (CD3^+^CD4^+^CD27^+^ CD45RO^-^), Central and Transitional Memory (T_CM_/T_TM_) (CD3^+^CD4^+^CD27^+^ CD45RO^+^), Effector Memory (T_EM_) (CD3^+^CD4^+^CD27^-^ CD45RO^+^) and Terminally Differentiated cells (T_TD_) (CD3^+^CD4^+^CD27^-^ CD45 RO^-^). Cells were stained with the apoptotic marker Annexin V and a viability dye. **A.** Gating strategy used to identify the following stages of cell death: live cells (Annexin V^-^ Viability^-^), early apoptotic cells (Annexin V^+^ Viability^-^), late apoptotic+necrotic cells (Annexin V^+^ Viability^+^) and total cell death (Annexin V^-^ Viability^+^). **B-C.** Percentage of cell death and apoptosis induced by the different single LRAs and their combinations in total CD4^+^ T cell population in presence (**B**) or absence (**C**) of the pan-caspase inhibitor Q-VD-OPh. **D-E.** Drug toxicities in different CD4^+^ T cell subpopulations, including T_NA_/T_SCM_, T_CM_/T_TM_, T_EM_ and T_TD_ in presence (**D**) or in absence (**E**) of Q-VD-OPh. Median values and min-max ranks are represented in panels B-E. In all panels, total dead cells are represented in green, early apoptosis is shown in orange and late apoptosis and necrosis is represented in blue.
(TIF)

S4 Fig Detection by the RNA FISH/flow assay of cells expressing HIV-RNA and p24 after viral reactivation in primary CD4^+^ T cells from HIV-infected patients. Isolated CD4^+^ T cells from 9 ART-suppressed HIV-infected individuals were reactivated with different LRAs for 22h and subjected to the RNA FISH/flow assay to analyze the frequency of cells expressing HIV-RNA and the viral protein p24. **A.** Gating strategy used to analyze HIV reactivation in CD4^+^ T cells and in the different CD4^+^ T cells subsets. **B.** Calculation of synergistic, antagonistic or additive effects in CD4^+^ T cells for the different combination of LRA families using the Bliss independence model. **C.** Percentage of cells expressing CD32^dim^ in HIV-1 RNA^+^ and HIV-1 RNA^-^ CD4^+^ T cells after treatment with the different LRAs plotted by Tukey boxplot. Medians of 9 independent experiments are shown in panels B and C. **D.** Correlation between the proportion of HIV-1 RNA^+^ cells per million cells, and the proportion of cells HIV-1 RNA^+^ expressing the viral protein p24. Spearman’s nonparametric correlation coefficient and associated P value are shown.
(TIF)

S5 Fig **A. Percentage of different CD4**^**+**^ **T cell subpopulations after treatment with the LRAs.** Percentage of each subset (T_NA_, T_SCM_, T_CM_, T_TM_, T_EM_ and T_TD_) was determined after 22 hours of culture with single or combination of LRAs (40 nM Romidepsin, 30 nM Panobinostat, 1 μM JQ1, 100 nM Ingenol, 10 nM Bryostatin-1, 81 nM PMA plus 1 μM Ionomycin or media alone) by flow cytometry. Dashed red line show the effect at 22h for the negative control, R10. Asterisks denote statistical significance compared with the negative control (R10) using a Friedman test followed by Dunn’s post hoc tests. *p<0.05, **p<0.01. **B.** Mean Fluorescence Intensity (MFI) of HIV-1 RNA^+^ cells after viral reactivation with Romidepsin (RMD), Ingenol (ING) and the combination of Romidepsin with Ingenol (RMD+ING).
(TIF)

S6 Fig Heatmaps and drug synergies in viral-reactivated CD4^+^ T cell subsets. **A-B.** Summary heatmaps of the potency of single LRAs (**A**) and their combinations (**B**) at increasing the proportion of HIV-1 RNA^+^ cells in the different CD4^+^ T cell subpopulations. **C.** Analysis of the interactions between LRAs, using the Bliss independence model, is shown for each CD4^+^ T cell subset. **D.** Interaction between LRAs on the ability to increase the proportion of p24^+^ cells within HIV-1 RNA^+^ cells in the T_CM_ subset are shown for each patient. Medians and min to max ranks are represented in panels C and D.
(TIF)

S1 Table Clinical data of patients included in the study. Patient ID, time since HIV diagnosis, CD4 cell count, % of CD4, viral load (cop/ml), time on suppressive ART and HAART regimen were included.
(PDF)
