# Supplementary figures and images for: Latency reversal agents affect differently the latent reservoir present in distinct CD4+ T subpopulations
Source: PLoS Pathog. 2019 Aug 19;15(8):e1007991. doi: 10.1371/journal.ppat.1007991 (PMC6715238; doi:10.1371/journal.ppat.1007991)

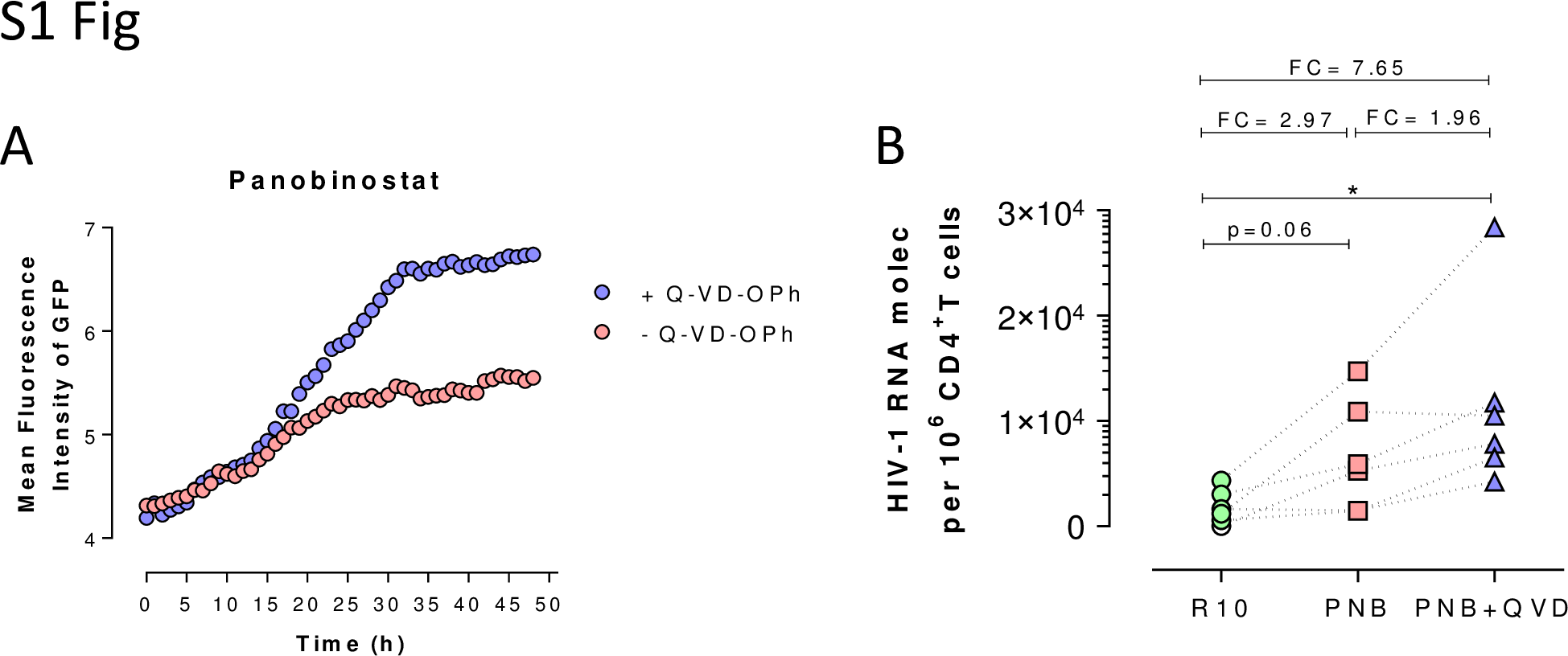

Supplement: S1 Fig — The effect of the pan-caspase inhibitor Q-VD-OPh on the detection of viral-reactivated cells was evaluated in isolated CD4+ T cells from ART-suppressed HIV-infected patients and in the latently infected cell line J-Lat (clone 10.6). A. GFP expression in J-Lat cells was monitored by the IncuCyte ZOOM live cell imaging system (Essen Bioscience) every hour for 50 hours after the addition of Panobinostat (30 nM) with or without Q-VD-OPh. B. CD4+ T cells were reactivated with Panobinostat (PNB, 30 nM) in the presence of Q-VD-OPh (10μM). Control cultures were treated with media alone (R10) or treated with media and Panobinostat (PNB). Copies of HIV-1 RNA per million CD4+ T cells were quantified in samples from 6 HIV+ patients by qPCR. Open circles show values under the limit of detection. Fold-change (FC) of viral reactivation is compared between conditions, but only in those where HIV-1 RNA was detected. (TIF) [file ppat.1007991.s001.tif]

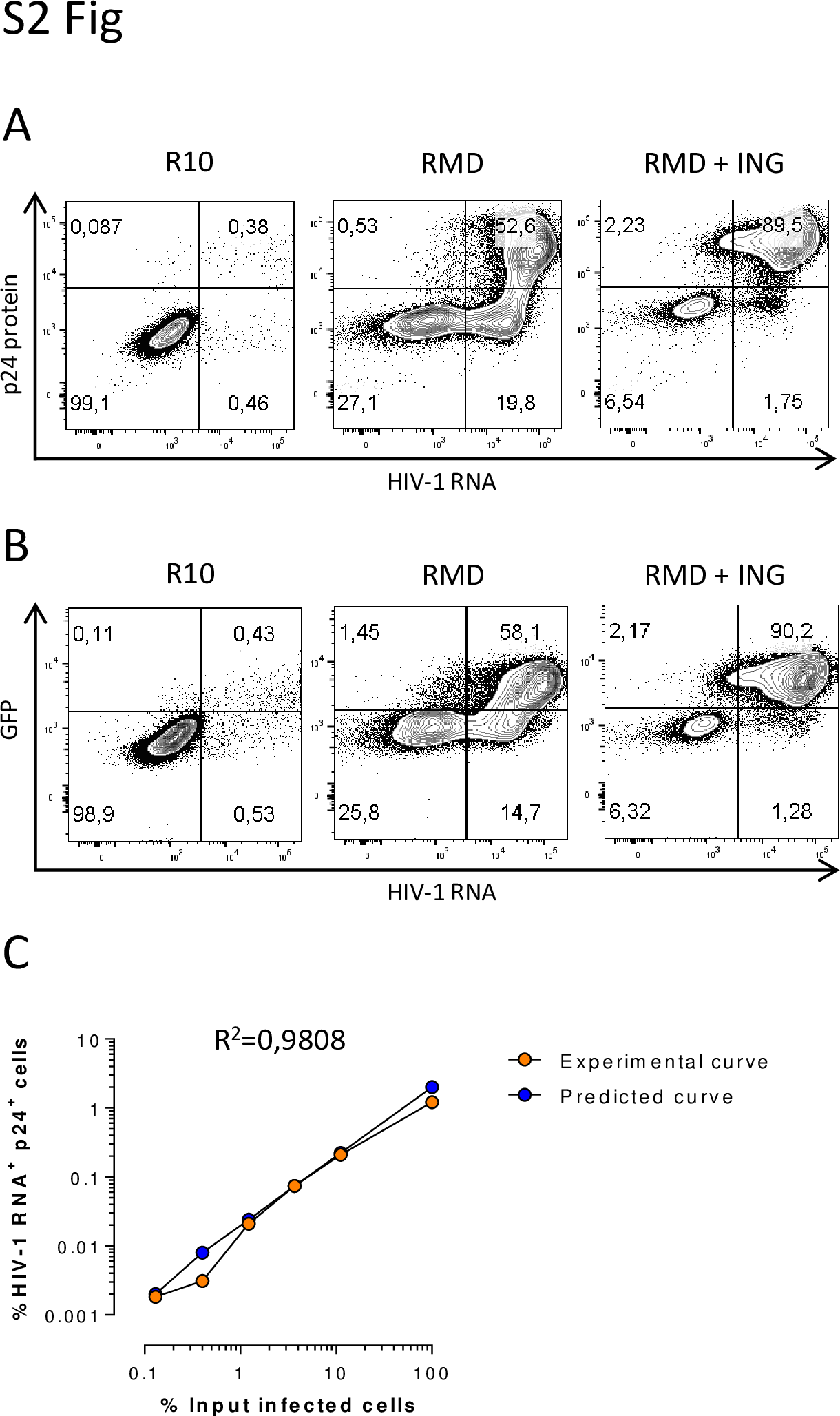

Supplement: S2 Fig — Cells were incubated for 22h with medium alone (R10), Romidepsin (RMD, 40 nM) or Romidepsin (40 nM) plus Ingenol (ING, 100 nM). Cells were then subjected to the RNA FISH/flow protocol and the proportion of HIV-1 RNA+ and p24+ (A) and HIV-1 RNA+ and GFP+ (B) cells was determined by flow cytometry. A flow cytometry plot for each condition is shown. C. Infection of primary CD4+ T cells from HIV-infected patients were expanded in vitro, and infected cells were diluted with uninfected cells to perform the quantification of predicted (blue symbols) versus experimental (orange symbols) values of HIV-1 RNA+ p24+ expression measured by the RNA FISH/flow assay. Assay linearity was assessed by linear regression. (TIF) [file ppat.1007991.s002.tif]

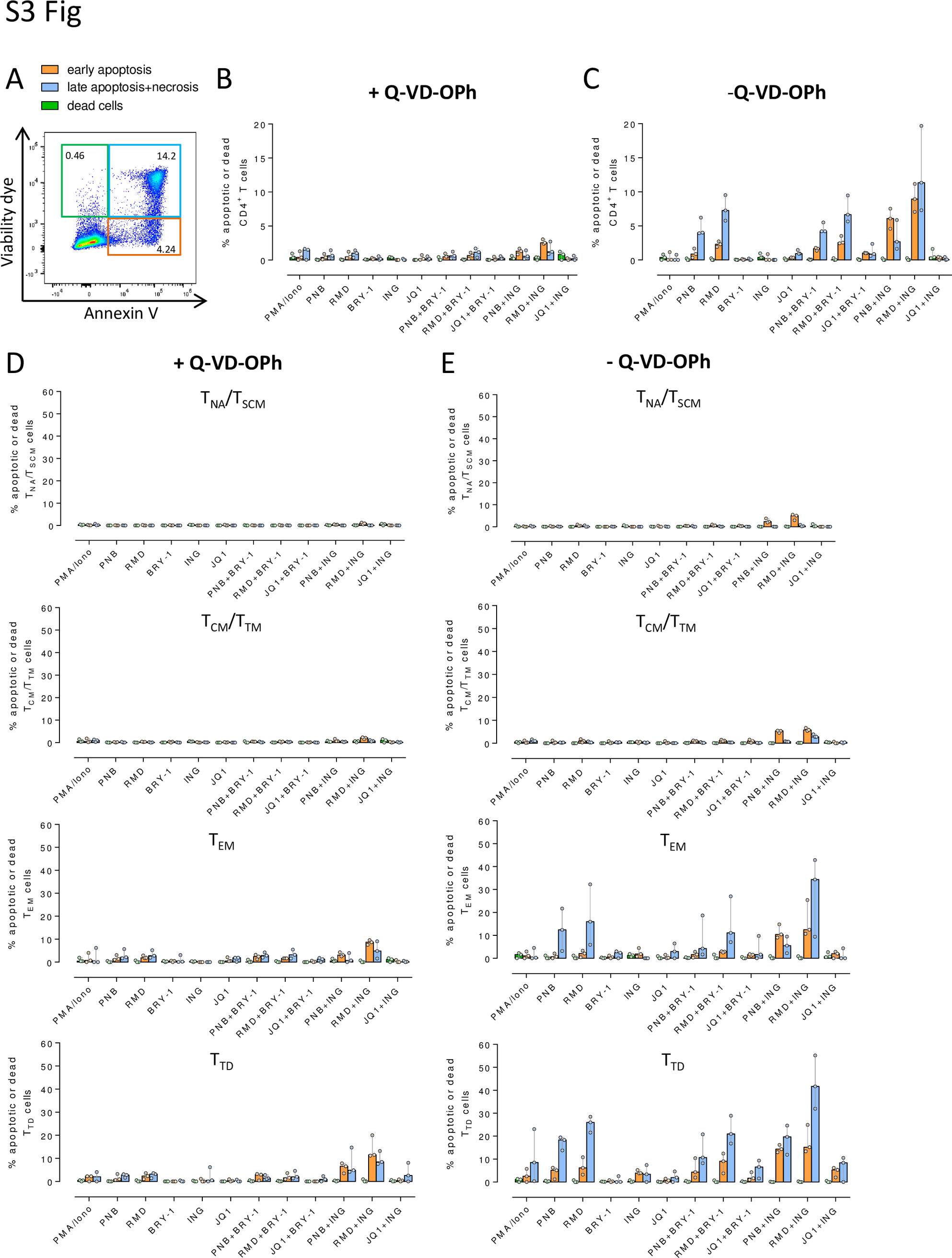

Supplement: S3 Fig — Isolated CD4+ T cells from 3 uninfected donors were incubated with the different drugs for 22 hours (40 nM Romidepsin, 30 nM Panobinostat, 1 μM JQ1, 100 nM Ingenol, 10 nM Bryostatin-1, 81 nM PMA plus 1 μM Ionomycin or media alone) and cell death was evaluated by flow cytometry in the whole CD4+ T cell population and in the different CD4+ T cell subsets. Cell subsets were identified as Naïve and Stem Cell Memory (TNA/TSCM) (CD3+CD4+CD27+ CD45RO-), Central and Transitional Memory (TCM/TTM) (CD3+CD4+CD27+ CD45RO+), Effector Memory (TEM) (CD3+CD4+CD27- CD45RO+) and Terminally Differentiated cells (TTD) (CD3+CD4+CD27- CD45 RO-). Cells were stained with the apoptotic marker Annexin V and a viability dye. A. Gating strategy used to identify the following stages of cell death: live cells (Annexin V- Viability-), early apoptotic cells (Annexin V+ Viability-), late apoptotic+necrotic cells (Annexin V+ Viability+) and total cell death (Annexin V- Viability+). B-C. Percentage of cell death and apoptosis induced by the different single LRAs and their combinations in total CD4+ T cell population in presence (B) or absence (C) of the pan-caspase inhibitor Q-VD-OPh. D-E. Drug toxicities in different CD4+ T cell subpopulations, including TNA/TSCM, TCM/TTM, TEM and TTD in presence (D) or in absence (E) of Q-VD-OPh. Median values and min-max ranks are represented in panels B-E. In all panels, total dead cells are represented in green, early apoptosis is shown in orange and late apoptosis and necrosis is represented in blue. (TIF) [file ppat.1007991.s003.tif]

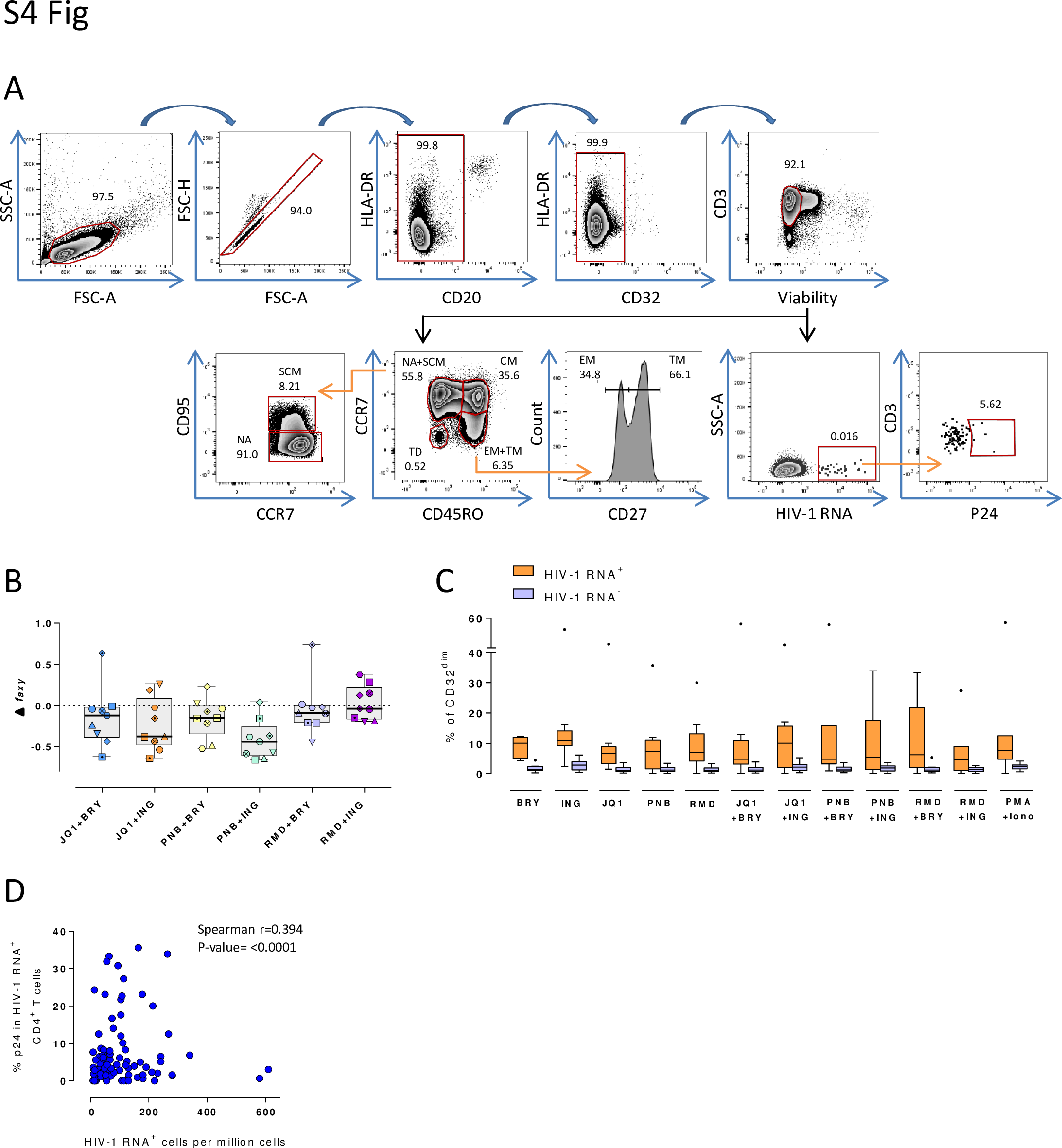

Supplement: S4 Fig — Isolated CD4+ T cells from 9 ART-suppressed HIV-infected individuals were reactivated with different LRAs for 22h and subjected to the RNA FISH/flow assay to analyze the frequency of cells expressing HIV-RNA and the viral protein p24. A. Gating strategy used to analyze HIV reactivation in CD4+ T cells and in the different CD4+ T cells subsets. B. Calculation of synergistic, antagonistic or additive effects in CD4+ T cells for the different combination of LRA families using the Bliss independence model. C. Percentage of cells expressing CD32dim in HIV-1 RNA+ and HIV-1 RNA- CD4+ T cells after treatment with the different LRAs plotted by Tukey boxplot. Medians of 9 independent experiments are shown in panels B and C. D. Correlation between the proportion of HIV-1 RNA+ cells per million cells, and the proportion of cells HIV-1 RNA+ expressing the viral protein p24. Spearman’s nonparametric correlation coefficient and associated P value are shown. (TIF) [file ppat.1007991.s004.tif]

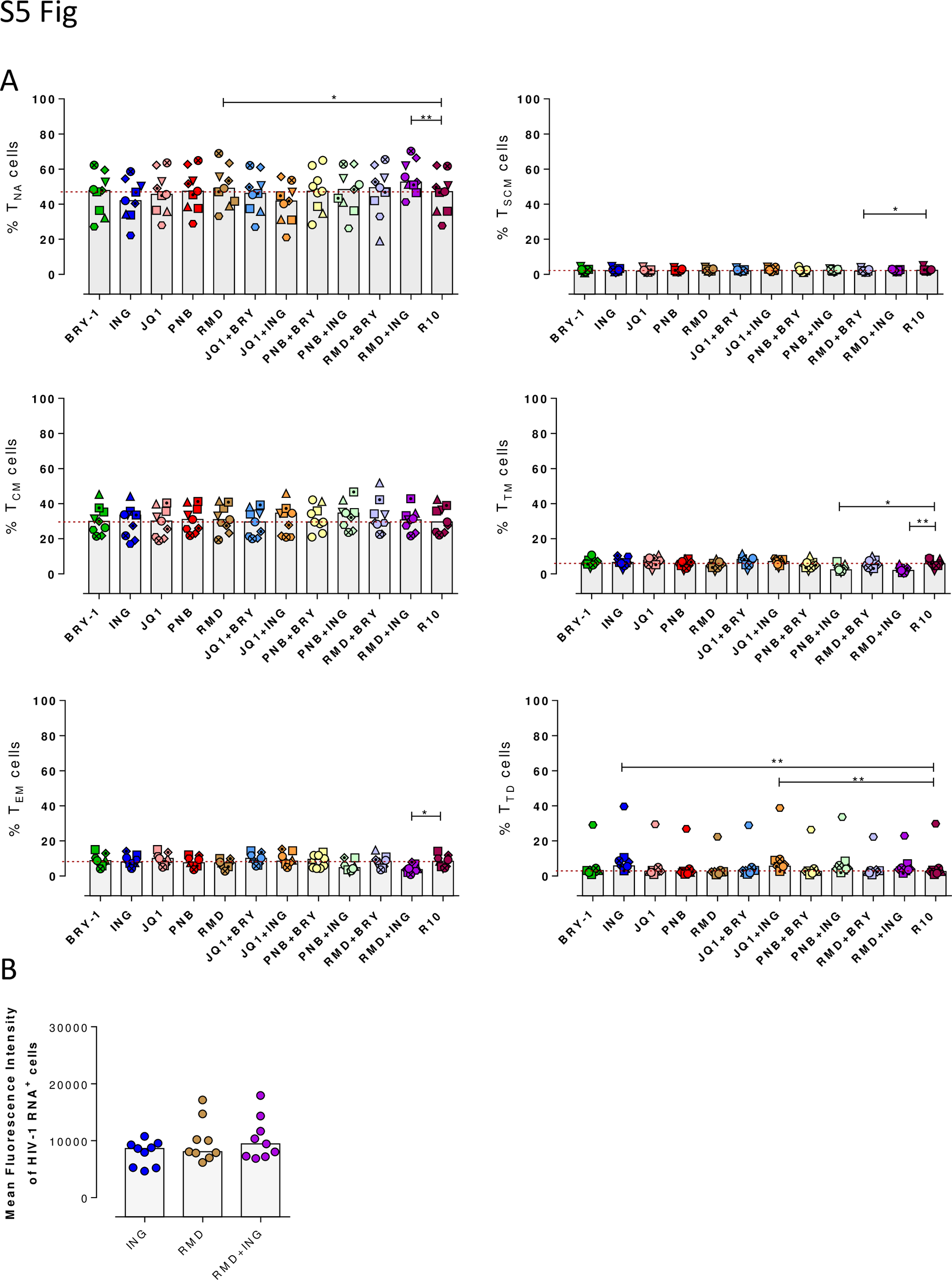

Supplement: S5 Fig — A. Percentage of different CD4+ T cell subpopulations after treatment with the LRAs. Percentage of each subset (TNA, TSCM, TCM, TTM, TEM and TTD) was determined after 22 hours of culture with single or combination of LRAs (40 nM Romidepsin, 30 nM Panobinostat, 1 μM JQ1, 100 nM Ingenol, 10 nM Bryostatin-1, 81 nM PMA plus 1 μM Ionomycin or media alone) by flow cytometry. Dashed red line show the effect at 22h for the negative control, R10. Asterisks denote statistical significance compared with the negative control (R10) using a Friedman test followed by Dunn’s post hoc tests. *p<0.05, **p<0.01. B. Mean Fluorescence Intensity (MFI) of HIV-1 RNA+ cells after viral reactivation with Romidepsin (RMD), Ingenol (ING) and the combination of Romidepsin with Ingenol (RMD+ING). (TIF) [file ppat.1007991.s005.tif]

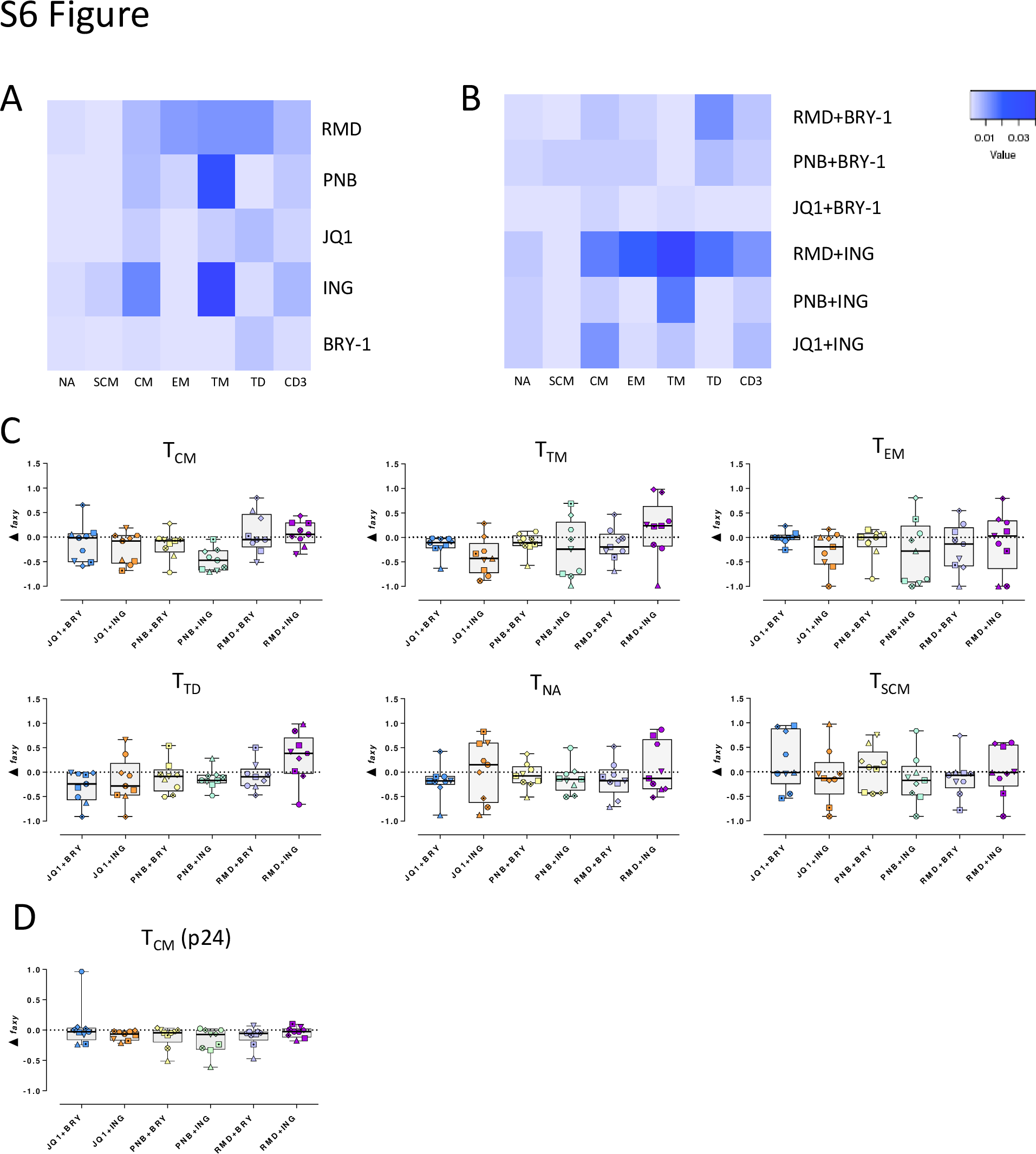

Supplement: S6 Fig — A-B. Summary heatmaps of the potency of single LRAs (A) and their combinations (B) at increasing the proportion of HIV-1 RNA+ cells in the different CD4+ T cell subpopulations. C. Analysis of the interactions between LRAs, using the Bliss independence model, is shown for each CD4+ T cell subset. D. Interaction between LRAs on the ability to increase the proportion of p24+ cells within HIV-1 RNA+ cells in the TCM subset are shown for each patient. Medians and min to max ranks are represented in panels C and D. (TIF) [file ppat.1007991.s006.tif]
