# Supplementary material for: Latency reversal agents affect differently the latent reservoir present in distinct CD4+ T subpopulations
Source: PLoS Pathog. 2019 Aug 19;15(8):e1007991. doi: 10.1371/journal.ppat.1007991 (PMC6715238; doi:10.1371/journal.ppat.1007991)
Supplement: S1 Table — Patient ID, time since HIV diagnosis, CD4 cell count, % of CD4, viral load (cop/ml), time on suppressive ART and HAART regimen were included. (PDF) [file ppat.1007991.s007.pdf]

S1 Table. Clinical data of patients included in the study.

Patient ID	Time since HIV diagnosis (months)	CD4 Cell Count (cells/µl)	%CD4	Viral Load (copies/ml)	Time on suppressive ART (months)	HAART regimen
**1**	72	800	23.12	<20	18	ABC/3TC+DTG
**2**	33	490	21.03	<20	28	TDF/FTC+EVG/c
**3**	31	560	27.92	<20	11	TDF/FTC+EVG/c
**4**	49	1070	38.14	<20	39	TDF/FTC/EFV
**5**	31	810	34.70	<20	22	ABC/3TC+DTG
**6**	42	1150	37.05	<20	37	ABC/3TC+RPV
**7**	13	1760	57.71	<20	>13	TDF/FTC+EVG/c
**8**	55	970	NA	<20	45	ABC/3TC+DTG
**9**	168	540	NA	<20	54	TDF/FTC+EVG/c
**10**	25	800	42.99	<20	19	TDF/FTC+EVG/c
**11**	35	1160	45.83	<20	31	TDF/FTC/EFV
**12**	276	520	29.50	<20	78	TDF/FTC+ETV
**13**	33	720	NA	<20	13	TDF/FTC+EVG/c
**14**	331	930	29.7	<20	71	TDF/FTC/EFV
**15**	127	960	45	<20	56	ABC/3TC+DTG
**16**	31	840	35.75	<20	23	TDF/FTC+EVG/c
**17**	18	1520	46.83	<20	6	ABC/3TC+ATV/r
**18**	48	850	40.64	<20	27	TDF/FTC+RPV

FTC, emtricitabine; TDF, tenofovir; 3TC, lamivudine; EFV, efavirenz; ABC, abacavir; EVG/c, elvitegravir boosted with cobicistat; DTG, dolutegravir; DRV/c, darunavir boosted with cobicistat; ETV, Etravirine; RPV, Rilpivirine; ATZ/r, atazanavir boosted with ritonavir; NA, not available.
